# Functional Characterization of the Grapevine γ-Glutamyl Transferase/Transpeptidase (E.C. 2.3.2.2) Gene Family Reveals a Single Functional Gene Whose Encoded Protein Product Is Not Located in Either the Vacuole or Apoplast

**DOI:** 10.3389/fpls.2019.01402

**Published:** 2019-11-04

**Authors:** Joshua G. Philips, Walftor Dumin, Christopher Winefield

**Affiliations:** Department of Wine Food and Molecular Biosciences, Faculty of Agriculture and Life Sciences, Lincoln University, Christchurch, New Zealand

**Keywords:** glutathione, γ-glutamyl transpeptidase, grape berry development, GGT, volatile thiol, New Zealand Sauvignon blanc

## Abstract

γ-glutamyl transferases/transpeptidases (E.C. 2.3.2.2, GGTs) are involved in the catabolism of many compounds that are conjugated to glutathione (GSH), which have a variety of roles. GSH can act as storage and transport vehicle for reduced sulfur; it is involved in the detoxification of xenobiotics and also acts as a redox buffer by utilizing its thiol residue to protect against reactive oxygen species, which accumulate in response to biotic and abiotic stress. Furthermore, many distinctive flavor and aroma compounds in Sauvignon blanc wines originate from odorless C5- and C6-GSH conjugates or their GGT catabolized derivatives. These precursors are then processed into their volatile forms by yeast during fermentation. In many plant species, two or more isoforms of GGTs exist that target GSH-conjugates to either the apoplast or the vacuole. A bioinformatics approach identified multiple GGT candidates in grapevine (*Vitis vinifera*). However, only a single candidate, VvGGT3, has all the conserved residues needed for GGT activity. This is intriguing given the variety of roles of GSH and GGTs in plant cells. Characterization of VvGGT3 from cv. Sauvignon blanc was then undertaken. The *VvGGT3* transcript is present in roots, leaves, inflorescences, and tendril and at equal abundance in the skin, pulp, and seed of mature berries and shows steady accumulation over the course of whole berry development. In addition, the *VvGGT3* transcript in whole berries is upregulated upon *Botrytis cinerea* infection as well as mechanical damage to leaf tissue. VvGGT3-GFP fusion proteins transiently over-expressed in onion cells were used to study subcellular localization. To confirm VvGGT3 activity and localization *in vivo*, the fluorescent γ-glutamyl-7-amido-4-methylcoumarin substrate was added to *Nicotiana benthamiana* leaves transiently over-expressing VvGGT3. In combination, these results suggest that the functional VvGGT3 is associated with membrane-like structures. This is not consistent with its closely related functionally characterized GGTs from *Arabidopsis*, radish and garlic.

## Introduction

In plants, glutathione (GSH, γ-Glu-Cys-Gly) is involved in a multitude of cellular processes. It is often conjugated by glutathione *S*-transferases (GSTs) to a wide range of chemicals, both derived from cellular metabolism and externally occurring xenobiotics (Rouhier et al., 2008). These GSH-conjugates are mostly targeted to the vacuole before being metabolized further (Edwards et al., 2000). The initial step of GSH-conjugate catabolism is carried out by γ-glutamyl transferases/transpeptidases (GGTs; EC 2.3.2.2), that promote the cleavage of the γ-glutamyl moiety of GSH. Following which, the remaining Cys-Gly-conjugated dipeptide is attacked by a proposed Cys-Gly dipeptidase to release Gly, with Cys remaining linked to the conjugate (Taniguchi and Ikeda, 2006; Castellano and Merlino, 2013; Masi et al., 2015).

In mammalian systems, where GGTs have been most extensively studied, GGTs are found to be N-terminally bound to the plasma membrane with the catalytic site facing the extracellular space. Following translation, mammalian as well as *Escherichia coli* GGTs undergo an autoproteolytic cleavage that is dependent on a conserved threonine residue (Hashimoto et al., 1995; Okada et al., 2006; Taniguchi and Ikeda, 2006) into a large and a small subunit. These subunits then self-assemble into functional heterodimers (Tate and Meister, 1978; Masi et al., 2007; Castellano and Merlino, 2013). GGT transcription is regulated in a tissue-specific manner and is highly represented in organs with secretory and absorptive functions such as the kidney and liver. GGTs play a role in maintaining cellular redox homeostasis by regulating the cellular levels of GSH (Castellano and Merlino, 2012).

In plants, there has been comparatively little research focus on GGTs. However, GGTs from a range of model as well as non-model plant species have begun to be functionally characterized (Nakano et al., 2006b; Yoshimoto et al., 2015). In *Arabidopsis thaliana* (*Arabidopsis*), where plant GGTs have been most extensively studied, four GGT genes have been identified, of which three are functional (*AtGGT1*, *AtGGT2*, and *AtGGT4*) (Grzam et al., 2007; Martin et al., 2007; Ohkama-Ohtsu et al., 2007a; Tolin et al., 2013). *AtGGT3* lacks segments of exons which possess catalytic activity and is most likely a pseudogene (Ohkama-Ohtsu et al., 2009). AtGGT1 and AtGGT2 are located in the apoplast, ionically-bound to the cell wall and/or plasma membrane, as they could only be liberated with high-salt treatments (Ohkama-Ohtsu et al., 2007a; Giaretta et al., 2017). Both are proposed to be involved in the prevention of oxidative stress by degrading the oxidized form of glutathione (GSSG) (Ohkama-Ohtsu et al., 2009; Noctor et al., 2012). *AtGGT1* is expressed throughout the plant with expression predominantly found in leaves and the vascular system, whereas *AtGGT2* is not transcribed in leaves but is specifically transcribed in GSH sink tissues such as seeds, pollen, and trichomes, *AtGGT2* is also weakly transcribed in roots (Martin et al., 2007; Destro et al., 2011; Giaretta et al., 2017). In *Atggt1* knock-out mutants, levels of GSSG in the apoplastic fraction from leaves are 7-fold higher than in wild type plants (Ohkama-Ohtsu et al., 2007a) indicating the need for AtGGT1 to metabolize GSSG. Phenotypically, *Atggt1* plants show yellowing in the older leaves. This is proposed to be caused by oxidative stress as indicated by higher lipid peroxidation in the mutant leaves (Martin et al., 2007; Ohkama-Ohtsu et al., 2007a). In a separate *Atggt1* knock-out study, Tolin et al. (2013) found both an increase in GSH content and a drop in the Cys-Gly content when compared to wild type plants, the authors accredit this to a loss of apoplastic GGT activity.

In *Atggt1/2* double knock-out mutants generated *via* RNAi, no significant changes to the overall levels of GSH were observed. However, the double mutant plants exhibited a reduction in seed yield and also produced fewer trichomes (where GSH levels were high) (Giaretta et al., 2017). *AtGGT4* is expressed in vacuoles and is involved in the degradation of GSH-conjugated xenobiotics (Grzam et al., 2007; Ohkama-Ohtsu et al., 2007b). It is divergent from the other three GGTs sharing only about 50% sequence identity with *AtGGT1* and is prevalent in the roots, where it accounts for 50% of total GGT enzyme activity (Grzam et al., 2007; Destro et al., 2011).

There are three functional GGT genes in radish, *RsGGT1* (BAC45233.1), *RsGGT2* (BAC56855.1), and *RsGGT3* (BAD22536.1). These genes have been used to assist in the classification of plant GGTs, which fall into two groups based on their solubility (either soluble or bound) (Nakano et al., 2006a; Nakano et al., 2006b). In addition, they have been used to classify GGTs based molecular structures, these include the monomeric GGT and heterodimeric GGT, both of which present different substrate specificities (Nakano et al., 2006a; Nakano et al., 2006b). Similarly, three functional GGT genes are present in garlic, *AsGGT1* (BAQ21911.1), *AsGGT2* (BAQ21912.1), and *AsGGT3* (BAQ21913.1) (Yoshimoto et al., 2015). AsGGT2 localizes to the vacuole, whereas AsGGT1 and AsGGT3 do not appear to possess a transit peptide targeting the protein for localization to intracellular organelles. In addition, the kinetic affinities differ based on both the γ-glutamyl donor substrate and the AsGGT enzyme used to catalyze the substrate (Yoshimoto et al., 2015).

New Zealand Sauvignon blanc wines have distinctive flavor and aroma, which are in part due to the volatile thiols, 3-mercaptohexan-1-ol (3MH, passion fruit) and its derivative 3-mercaptohexyl-acetate (3MHA, boxwood) (Lund et al., 2009). Other volatile thiols present in Sauvignon blanc wines include 4-mercapto-4-methyl-2-pentanone (4MMP, boxwood) and 4-mercapto-4-methyl-2-pentanol (4MMPOH, citrus zest) (Darriet et al., 1995; Tominaga et al., 1998; des Gachons et al., 2000). These C5 and C6 based compounds have been discovered in free form in most tropical fruits, but not in grape berries, where they are found as odorless GSH, Cys-Gly, and Cys- coupled precursors (Pena-Gallego et al., 2012). A pathway for the biosynthesis of the volatile thiol 3MH has been proposed by Kobayashi et al. (2011), involving the conjugation of C6 hexenal to GSH to produce 3MH-GSH (3MH-*S*-glut). The breakdown of 3MH-*S*-glut forming other recognized precursors is proposed to involve the action of GGT to produce 3MH-*S*-Cys-Gly, as well as additional enzymes that include carboxypeptidases to produce 3MH-*S*-Cys. It is now considered that these volatile precursors are released by the action of yeasts during the alcoholic fermentation of grape (Pena-Gallego et al., 2012).

Pathogen infections are thought to stimulate stress response pathways *via* the production of pathogen-associated molecular patterns and/or elicitors during the fungal infection (Thibon et al., 2009; Thibon et al., 2011). These responses, in turn, upregulate the production of compounds that are able to be conjugated by GSH and grape berries infected with *Botrytis cinerea* have much higher levels of 3MH-*S*-Cys (Thibon et al., 2009; Thibon et al., 2011). Kobayashi et al. (2011) provided data on the involvement of GSTs and GGTs in the formation of volatile thiol precursors in grapes. However, other than transcript and GGT enzyme activity data in grape berries exposed to heat and UV-C stress, no information on the wider roles for grape GGT currently exists.

Grapevine appears to contain only a single GGT gene, possessing all the key conserved residues needed for GGT activity (annotated as VvGGT3, NCBI accession XP_002280190.1). This raises many questions about the role of this enzyme compared to other plants, where at least two GGTs exist, and specifically with respect to its role in the formation of important flavor and aroma precursors in grape berries. Consequently, we set out to functionally characterize VvGGT3 from *V. vinifera* L. cv. Sauvignon blanc. We explored subcellular localization as well as *VvGGT3* transcript abundance in a range of tissues, including whole grape berry development and whole berries infected with *Botrytis*.

## Materials and Methods

### Plant Material

*V. vinifera* L. cv. Sauvignon blanc whole berries for gene expression studies, as described in Podolyan et al. (2010), were collected at 20, 30, 50, 60, 80, 90, 100, and 110 days after anthesis (daa). Collection was during development in the 2006, 2007, 2008, and 2009 seasons from the Booker vineyard, Brancott Estate, Marlborough, New Zealand (41°.56′ South, 173°.85′ East). Berries harvested at 110 daa had soluble solid levels of between 21 and 23 °Brix and were considered fully ripe. For each time point, the phenological stages mostly represented E-L 30, 31, 32, 35, 36, 36, 37, and 38, respectively (Coombe, 1995). Root tip (∼15 mm), leaf (∼225 mm^2^), E-L 15 inflorescence (Coombe, 1995), and tendril (∼20 mm), as described in Tashiro et al. (2016), were also collected from the Booker vineyard during dormancy in 2010 and grown as single node cuttings in the Lincoln University glasshouse nursery, Canterbury, New Zealand (43°.38′ South, 172°.27′ East) in 2011. Young leaves (∼2.5–3 cm wide) used for wounding responses were also collected from the Lincoln University glasshouse nursery in 2013. Skin, pulp and seed fractions were separated from mature whole Sauvignon blanc grape berries at 21 °Brix and *B. cinerea-*infected berries were collected from the Lincoln University research vineyard in the 2012 and 2014 seasons, respectively. Uninfected berries on the same bunch had a soluble solids content of 21 °Brix, mostly representing E-L 38 berries (Coombe, 1995). All samples were collected in biological triplicates and were snap-frozen in liquid nitrogen then stored at -80°C until use. The LAB genotype of *Nicotiana benthamiana* (Bally et al., 2018) was used in transient transformation experiments. These plants were grown in a controlled environment room at Lincoln University until 4 weeks of age, at constant temperature of 25°C with a 16/8 h photoperiod and a light intensity of 180 µM · m^−2^ · s^−1^. Onion biolistic transient transformation experiments were carried out using red onion (*Allium cepa* L., family Amaryllidaceae) bulbs purchased locally.

### RNA Extraction and cDNA Synthesis

The triplicate samples of the various tissue types were ground in liquid nitrogen and ∼100 mg was processed by the Spectrum Plant Total RNA kit (Sigma-Aldrich). The resultant total RNA, ∼300 ng, was reverse transcribed by PrimeScript RT reagent Kit (Perfect Real Time, TaKaRa Bio Inc.) in a 10 µL reaction and then diluted to a final volume of 200 µL, as described in Tashiro et al. (2016). For later stage berries (100 and 110 daa), ∼200 mg of ground tissue was used and supplemented with a proportional increase in buffers. For seed tissue, 5% w/v Polyvinylpolypyrrolidone (Sigma-Aldrich) was added to the lysis buffer.

### Isolation and Cloning of *VvGGT3*

The *VvGGT3* open reading frame was amplified from Sauvignon blanc grape berry cDNA using VvGGT3 gateway forward and VvGGT3 reverse primers (**Supplementary Table 1**). Amplification was carried out using PrimeStar HS DNA polymerase (TaKaRa Bio Inc.) according to manufacturer's instructions. The PCR amplification was conducted using the following conditions; 30 cycles comprising a denaturation step at 98^°^C for 10 s, annealing at 55^°^C for 5 s and extension at 72^°^C for 2 min. The VvGGT3 PCR fragment was subsequently cloned into pENTR/D-TOPO (ThermoFisher), according to manufacturer's instructions. DNA isolated from individual clones was sequenced to confirm the identity of VvGGT3 and this sequence has been deposited to NCBI (VvGGT3_ORF; MN101215.3).

*VvGGT3* was re-amplified with VvGGT3 gateway forward and the VvGGT3 reverse-STOP primers (**Supplementary Table 1**) and this amplicon was subcloned into pB7FWG2 (Karimi et al., 2002) for subcellular localization. A shorter VvGGT_N75_ fragment was also subcloned into pB7FWG2 using the VvGGT3 (N75) gateway forward and VvGGT3 (N75) reverse primers (**Supplementary Table 1**). To explore *in vivo* VvGGT3 enzymatic activity, *VvGGT3* was subcloned into pEAQ-HT-DEST2 (Sainsbury et al., 2009) using Gateway cloning as per manufacturer's instructions (ThermoFisher). Each of the binary-destination vectors containing *VvGGT3* was checked for integrity by sequencing and finally transferred into *Agrobacterium tumefaciens* GV3101 (MP90) by electroporation, using a MicroPulser (BioRad), with the *Agrobacterium* setting.

### Transient Transformation of Red Onion Epidermal Cells and *N. benthamiana* Leaves Using Biolistics and *Agrobacterium*-Mediated Transformation, Respectively

Transient transformation of the inner epidermis of excised bulb leaves of red onion and *N. benthamiana* leaves with VvGGT3 and positive GFP control constructs was carried out according to Wiltshire et al. (2017) and Dumin et al. (2018), respectively. A positive eGFP control construct was obtained by liberating 3×eGFP-Hyg^R^ as a *SfiI* restriction fragment from pUMA647 (Scherer et al., 2006). This fragment was then placed under the transcriptional control of the CaMv35S promoter in pBS-SK (Artemio Mendoza, unpublished data). For transient transformations of onion inner epidermal cells, purified plasmid constructs were deposited onto gold particles and used to bombard the inner epidermis of excised bulb leaves using an particle inflow gun (Kiwi Scientific, Levin, New Zealand) as described in Wiltshire et al. (2017). Leaf pieces were incubated for 24 h prior to imaging. GFP fluorescence images were obtained using confocal microscopy (Zeiss LSM510, Axio imager, with a LCI plan-neofluar 25×0.8 Imm corr DIC objective). Images were captured using the fluorescence contrast method with an excitation wavelength of 488 nm, an emission wavelength of 538 nm and a pinhole of 3.86 AU/78 µm.

Transient transformation of *N. benthamiana* leaves was performed according to Dumin et al. (2018). Briefly, overnight cultures of transformed *Agrobacterium* were centrifuged, the pellets washed with half-strength Murashige and Skoog medium (Duchefa Biochemie), re-suspended to an OD_600_ of 0.2 and acetosyringone (Sigma-Aldrich) added to a final concentration of 100 µM. The re-suspended cultures were subsequently incubated at room temperature on a rocking platform for at least 1 h prior to being used for transient agro-infiltrations. Infiltrated leaves were left on the plant for either: 3 days prior to excision for GFP analysis at 30% laser power, 501–551 nm (green color) wavelengths, using the TCS SP5 confocal microscope (Leica Microsystems), as described in Dumin et al. (2018) or 5 days for *in vivo* activity. At least 10 individual infiltrations were prepared for each experiment and representative images presented.

### *In vivo* Confirmation of VvGGT3 Enzyme Activity

The metabolism of a fluorescent γ-GGT substrate was carried out as per Storozhenko et al. (2002). After 5 days post agro-infiltration, leaf discs from at least 10 independent infiltrations were cut and vacuum infiltrated twice for 2 min (Lizamore and Winefield, 2015) with a solution containing 0.1 M Tris-HCl [pH 7.5], 1 mM γ-glutamyl-7-amido-4-methylcoumarin (Biosynth), and 40 mM glycylglycine (Sigma-Aldrich). The leaf discs were then briefly rinsed in molecular grade water and immediately mounted onto microscope slides before being examined at 10x magnification using a DM IRB inverted fluorescent microscope (Leica), with excitation at 340–380 nm and emission at 425 nm. Images were captured using SPOT Software v4.6 (Diagnostic Instruments Inc.). Negative controls consisted of leaves transiently transformed with *Agrobacterium* GV3101 (MP90) without a transgene and treated in exactly the same manner as described.

### Transcript Profiling of *VvGGT3* in Grape Tissues

Quantitative reverse-transcriptase PCR (RT-qPCR) was performed on cDNA synthesized as described earlier on the triplicate samples. Assays were designed and executed according to MIQE guidelines (Bustin et al., 2009). Each individual PCR reaction was carried out with triplicate technical replication for each biological replicate. *VvActin* and *VvEF1α* were described as being suitable normalization factors in grape berry (Reid et al., 2006). We thus used *VvActin* as described in Reid et al. (2006) and an optimized *VvEF1α* qPCR primer set, as described Tashiro et al. (2016), as normalization factors. Relative expression assays were run using the Eco Real-Time PCR System (Illumina) and resulting data analyzed with the supplied Illumina Eco software. Final reaction volumes were 10 µL and are as described in Philips et al. (2019), with primer sequences presented in (**Supplementary Table 1**). All liquid handling steps were performed using an Eppendorf epMotion 5070 liquid handling robot. PCR efficiency, standard curves, and no-template controls were determined and confirmed as described in Tashiro et al. (2016). The qRT-PCR conditions consisted of; 95°C for 5 min, followed by 2-step PCR of 40 cycles comprising of 95°C for 5 s and 60°C for 30 s, with fluorescence being recorded during the 60°C extension step. Following this, one cycle of melt curve analysis was performed for each amplified product as per default Illumina Eco settings.

### Phylogenetic Analysis

Two multiple alignments were performed on the selection of protein sequences retrieved (**Table 1**) using the default ClustalW settings within Geneious 10.1.3 (Biomatters Ltd.) and consisted of the BLOSUM62 cost matrix, gap open cost of 10 and gap extend cost of 0.1. The first alignment included full length plant GGTs, where V77 of VvGGT3 is the first largely conserved (97.7%) residue. The second alignment excluded the N-terminal leader and targeting sequences by removal of residues prior to this position. Cladograms were then generated from both the full length and truncated alignments using the default tree builder feature within Geneious 10.1.3 (Biomatters Ltd.); these include Jukes-Cantor as the genetic distance model and the tree built with the neighbor-joining method with no outgroup. The multiple protein sequence alignments of both the full length and truncated GGTs are presented in **Supplementary Data Sheet 2** and **3**.

**Table 1:** List of plant GGT enzymes and their accessions used to generate the phylogenetic tree presented in Figure 1. GGTs associated with a reference have been completely or partially characterized.

Organism	Common name	Gene	NCBI accession	Reference
*Allium cepa*	Onion	Onion GGT	AAL61611.2	Lancaster and Shaw (1994); Shaw et al. (2005); Sun et al. (2019)
*Allium sativum*	Garlic	Garlic GGT1	BAQ21911.1	Ceci et al. (1992); Cho et al. (2012); Yoshimoto et al. (2015); Sun et al. (2019)
		Garlic GGT2	BAQ21912.1	
		Garlic GGT3	BAQ21913.1	
*Ananas comosus*	Pineapple	Pineapple GGT1	OAY79948.1	
		Pineapple GGT3	XP_020102589.1	
*Arabidopsis thaliana*	*Arabidopsis*	*Arabidopsis* GGT1	NP_195674.2	Storozhenko et al. (2002); Grzam et al. (2007); Martin et al. (2007); Ohkama-Ohtsu et al. (2007a); Ohkama-Ohtsu et al. (2007b); Destro et al. (2011); Tolin et al. (2013); Giaretta et al. (2017)
		*Arabidopsis* GGT2	NP_195675.2	
		*Arabidopsis* GGT4	NP_194650.1	
*Capsella rubella*	*Capsella*	*Capsella* GGT1	XP_006283415.1	
		*Capsella* GGT2	XP_006285909.1	
		*Capsella* GGT3	XP_006283327.1	
*Cicer arietinum*	Chickpea	Chickpea GGT1	XP_004502464.1	
		Chickpea GGT3	XP_004513075.1	
*Citrus clementina*	Clementine	Clementine GGT1*	XP_006428086.1	
		Clementine GGT3	XP_006451358.1	
		Clementine GGT3 X1*	XP_006451357.1	
*Citrus sinensis*	Sweet orange	Sweet orange GGT1	XP_006464312.1	
		Sweet orange GGT3	XP_006475363.1	
		Sweet orange GGT3 X1*	XP_006475362.2	
*Cucumis sativus*	Cucumber	Cucumber GGT1	XP_004143492.1	
		Cucumber GGT3	XP_004141035.1	
*Daucus carota* subsp. *sativus*	Carrot	Carrot GGT1	XP_017229362.1	
		Carrot GGT3	XP_017240209.1	
		Carrot GGT3 X1*	XP_017223556.1	
*Elaeis guineensis*	African oil palm	African oil palm GGT1	XP_010905955.1	
		African oil palm GGT3	XP_010920650.1	
*Fragaria vesca*	Strawberry	Strawberry GGT1	XP_004302214.1	
		Strawberry GGT3	XP_004287500.1	
*Glycine max*	Soybean	Soybean GGT1	XP_003552129.1	
		Soybean GGT1 X1*	XP_003538504.1	
		Soybean GGT3	XP_003516303.1	
		Soybean GGT3 X1	XP_003529066.1	
*Hordeum vulgare*	Barley	Barley GGT1*	BAK03021.1	Ferretti et al. (2009)
		Barley GGT3*	HORVU3Hr1G013650.5	
*Juglans regia*	English walnut	English walnut GGT1	XP_018838119.1	
		English walnut GGT3	XP_018846370.1	
		English walnut GGT3 X1	XP_018846551.1	
*Malus domestica*	Apple	Apple GGT1	XP_008387030.1	
		Apple GGT3	XP_008387437.1	
		Apple GGT3 X1*	XP_017178339.1	
*Manihot esculenta*	Cassava	Cassava GGT1 X1	XP_021633816.1	
		Cassava GGT3	XP_021608812.1	
		Cassava GGT3 X1	XP_021597481.1	
*Medicago truncatula*	Barrel medic	Barrel medic GGT1*	XP_003602023.2	
		Barrel medic GGT3*	XP_003620774.1	
*Morus notabilis*	Mulberry	Mulberry GGT1	XP_024023634.1	
		Mulberry GGT3	XP_010086906.2	
*Musa acuminata* subsp. *malaccensis*	Banana	Banana GGT1	XP_009401074.1	
		Banana GGT3	XP_009404605.1	
		Banana GGT3 X1*	XP_009405839.1	
*Nicotiana benthamiana*	*Nicotiana benthamiana*	*N. benthamiana* GGT1	Nbv5.1tr6222694	
		*N. benthamiana* GGT3	Nbv5.1tr6205538	
		*N. benthamiana* GGT3 X1*	Nbv5.1tr6236734	
*Nicotiana tabacum*	Tabacco	Tobacco GGT1	XP_016466580.1	Steinkamp and Rennenberg (1984); Nakano et al. (2004)
		Tobacco GGT3	XP_016477075.1	
		Tobacco GGT3 X1*	XP_016472806.1	
*Oryza sativa* Japonica Group	Japanese rice	Japanese rice GGT1	XP_015633796.1	
		Japanese rice GGT3	XP_015629579.1	
		Japanese rice GGT3 X1*	XP_015618688.1	
*Phaseolus vulgaris*	Common bean	Common bean GGT1*	XP_007163614.1	
		Common bean GGT3*	XP_007152946.1	
*Phoenix dactylifera*	Date palm	Date palm GGT1	XP_017700228.1	
		Date palm GGT3 X1	XP_008784503.1	
*Populus trichocarpa*	Poplar	Poplar GGT1	XP_002298608.3	
		Poplar GGT1 X1	XP_024458179.1	
		Poplar GGT3	XP_024446158.1	
*Prunus mume*	Japanese apricot	Japanese apricot GGT1	XP_008234476.1	
		Japanese apricot GGT3	XP_008242458.1	
*Prunus persica*	Peach	Peach GGT1	XP_020421714.1	
		Peach GGT3	XP_020424698.1	
*Pyrus x bretschneideri*	Chinese white pear	Chinese white pear GGT1	XP_009372517.1	
		Chinese white pear GGT3	XP_009352585.1	
*Raphanus sativus*	Radish	Radish GGT1	BAC45233.1	Nakano et al. (2004); Nakano et al. (2006a); Nakano et al. (2006b)
		Radish GGT2	BAC56855.1	
		Radish GGT3	BAD22536.1	
*Sesamum indicum*	Sesame	Sesame GGT1 X1	XP_011090592.1	
		Sesame GGT3 X1	XP_011093665.1	
*Solanum lycopersicum*	Tomato	Tomato GGT1	XP_004240001.1	Martin and Slovin (2000); Sorrequieta et al. (2010)
		Tomato GGT3	XP_004251649.1	
*Solanum tuberosum*	Potato	Potato GGT1	XP_006355534.1	
		Potato GGT3	XP_006353508.1	
*Sorghum bicolor*	*Sorghum*	*Sorghum* GGT1	XP_002447955.2	
		*Sorghum* GGT3	XP_002457361.1	
*Theobroma cacao*	Cocoa	Cocoa GGT1	XP_007048081.2	
		Cocoa GGT3	XP_017982879.1	
		Cocoa GGT4	EOY30584.1	
*Vitis vinifera*	Grape	Grape GGT3 (VvGGT3)	XP_002280190.1	This study
*Zea mays*	Maize	Maize GGT1	NP_001147571.1	Masi et al. (2007)
		Maize GGT3	XP_020398122.1	

*Assigned nomenclature of predicted GGT proteins as determined by sequence similarity and hence presumed function.

Unless otherwise stated; plant GGT sequences were retrieved from https://www.ncbi.nlm.nih.gov/, accessed 20/04/2018.

N. benthamiana sequences were retrieved from http://benthgenome.qut.edu.au/, accessed 18/08/2016.

Barley GGT3* sequence was retrieved from http://ensembl.gramene.org/Hordeum_vulgare/Info/Index, accessed 19/07/2017.

### Statistical Analysis

Post-hoc analysis of the mean relative abundance of *VvGGT3* in the various tissue types and treatments was performed by the Tukey-Kramer test after ANOVA using GenStat v15 (VSN International, Ltd.). Means significantly different at *P* < 0.05 are indicated by different letters above each bar in the graphs.

## Results

### Vitis GGT Isoforms and Phylogenetic Analysis

Initial mining of the 12X grape genome reference sequence deposited on NCBI, accessed 22/11/2016 revealed a single GGT sequence, (XP_002280190.1, designated as VvGGT3), which is unlike most other plant species where multiple genes encode GGT isoforms (**Table 1**). These GGT sequences from planta, retrieved from online repositories, are either published and characterized or currently putative. Since then, mining the latest 12X.v2 genome (http://genomes.cribi.unipd.it/grape/, accessed 30/08/2019) revealed three GGT candidates. The first on chromosome 11 has three splice variants (VIT_211s0016g02830.1,.2, and.3), encoding putative GGTs at 626, 475, and 557 amino acids (AA) in length, respectively. The other potential GGTs, VIT_212s0142g00530, located on chromosome 12, and VIT_201s0146g00200, located on chromosome 1, are 406 and 174 AA in length, respectively. In addition, mining UniProt (accessed 30/08/2019), reveals two potential GGTs. The first, designated D7TBD5 is identical in sequence to XP_002280190.1 and VIT_211s0016g02830.1. The second, A0A438IHM7, is slightly larger at 683 AA in length.

**Figure 1:**
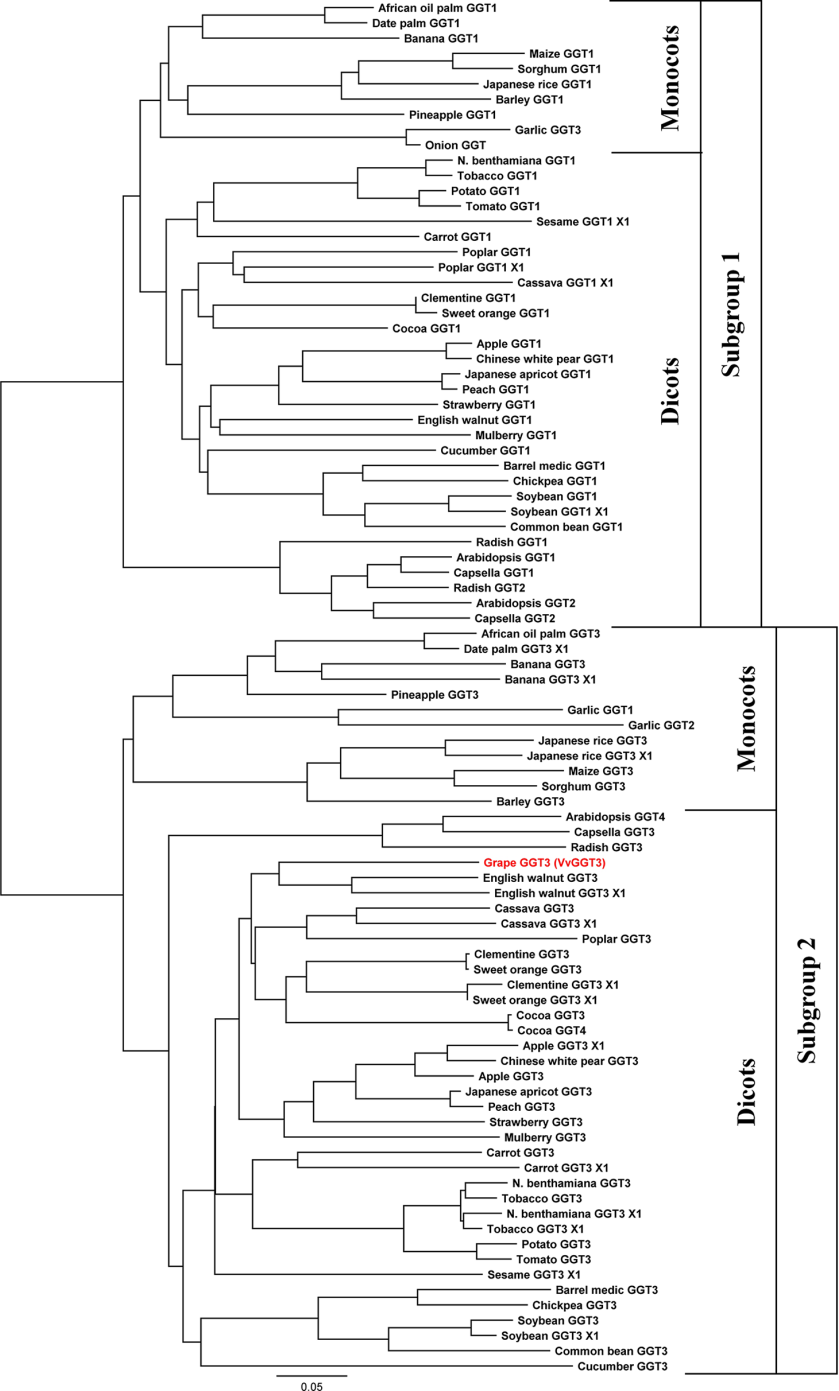
Phylogeny of selected plant γ-glutamyl transferases/transpeptidases (GGTs). The accession numbers of these GGTs are presented in **Table 1**. GGTs group into two distinctive subgroups, then further into the monocot and dicot clades. Subgroup 1 has GGTs, which are thought to prevent oxidative stress by degrading the oxidized form of glutathione (GSSG) and the breakdown of extracellular GSH in the apoplast. Subgroup 2 has GGTs which are thought to degrade GSH-conjugates in the vacuole. The GGT from grape, VvGGT3, highlighted in red, falls under subgroup 2. Multiple sequence alignments of deduced protein sequence that were used in this analysis can be found in **Supplementary Data Sheet 2**.

Generation of a phylogenetic tree with the deduced VvGGT3 amino acid sequence (XP_002280190.1) with a selection of deduced plant GGT amino acid sequences revealed that when two or more genes for GGT were present in a species that they grouped into two distinct subgroups (**Figure 1**). In accordance to the functional work carried out in *Arabidopsis* and garlic, these two subgroups possess GGTs that target either the apoplast or the vacuole (Yoshimoto et al., 2015; Giaretta et al., 2017). Based on the phylogenetic analysis, VvGGT3 groups with *Arabidopsis* GGT4 (AtGGT4) and garlic GGT2 (AsGGT2) both of which have been functionally shown to be located in the vacuole (Grzam et al., 2007; Yoshimoto et al., 2015). Additionally, radish has three at least GGT enzymes (Nakano et al., 2006b) of which RsGGT3 is the closest match to VvGGT3 (**Figure 1**). When over-expressed in tobacco, RsGGT3 unlike RsGGT1 and RsGGT2 which are most likely bound to cell walls was able to be purified from the soluble fraction (Nakano et al., 2006b), suggesting that it too like AtGGT4 may be localized to the vacuole (Ohkama-Ohtsu et al., 2009). Removal of the putative N-terminal leader/targeting peptides and reconstruction of the phylogenetic tree does not substantially affect the sequence relationships observed (**Supplementary Figure 1**).

Generation of a phylogenetic tree with the other potential *Vitis* GGT candidates was then undertaken (**Supplementary Figure 2**). As expected, the splice variants, VIT_211s0016g02830.2, .3, and UniProt A0A438IHM7 clustered together with VvGGT3 (XP_002280190.1). Surprisingly, none of the candidates grouped to subgroup 1. Candidate *GGT* genes, VIT_212s0142g00530 and VIT_201s0146g00200, were the most divergent when compared to the other plant GGT sequences (**Supplementary Figure 2**).

A subset of the functionally characterized plant GGTs, along with *Escherichia coli*, human, and rat GGTs, where key residues have been characterized, were then aligned with the potential *Vitis* GGT candidates (**Supplementary Figure 3**). Compared to other GGTs, the UniProt A0A438IHM7 candidate has a longer N-terminus sequence, most likely from mis-annotation of the genome. Key residues, essential for GGT activity are conserved in bacterial, mammalian, and plant GGT sequences (**Supplementary Figure 3**). These residues include: R107 and D423 in human, which are involved in the binding of substrates (Taniguchi and Ikeda, 2006); the catalytic nucleophile, T391 in *E. coli*, and the residue involved in stabilizing the nucleophile, T409 (Okada et al., 2006); residues S451 and S452 in human, which are involved in enzyme catalysis (Okada et al., 2006; Taniguchi and Ikeda, 2006); and G483 and G484 in *E. coli*, which comprise the GGT oxyanion hole (Okada et al., 2006). Of all the VvGGT isoforms, only the R107 residue in human is conserved, apart from splice variant VIT_211s0016g02830.3, where it is missing. The VIT_212s0142g00530 and VIT_201s0146g00200 candidates, do not possess the remaining conserved residues. Furthermore, they are also missing the region that has been described as the GGT molecular signature, [T-[STA]-H-x-[ST]-[LIVMA]-x(4)-G-[SN]-x-V-[STA]-x-T-x-T-[LIVM]-[NE]-x(1,2)-[FY]-G] (Ferretti et al., 2009) (**Supplementary Figure 3**). As the VIT_212s0142g00530 and VIT_201s0146g00200 GGT candidates are deficient in the key GGT residues, molecular signature and lack the length of other plant GGTs, they are not considered to be genes possessing "classical" GGT activity.

Separation of the PCR amplification of *VvGGT3* from Sauvignon blanc berry cDNA as described in *Materials and Methods*, produced a single distinct band, which was sequenced to be the transcript encoding the 626 AA variant (XP_002280190.1 and VIT_211s0016g02830.1). The nucleotide sequence in cv. Sauvignon blanc has been deposited to NCBI (MN101215.3). Splice variant VIT_211s0016g02830.2, is missing the first conserved residue, R107 (in human) needed of GGT substrate binding (Taniguchi and Ikeda, 2006). Whereas, splice variant VIT_211s0016g02830.3 does not have conserved residues at its C-terminus, when compared to the selection of other plant GGT sequences (**Supplementary Figure 3**). It is therefore likely that these splice variants are mis-annotated. Due to the aforementioned reasons and as we were unable to experimentally detect the other splice variants, we consider cv. Sauvignon blanc as possessing a sole *GGT* gene.

### Functional Characterization of VvGGT3

**Figure 2:**
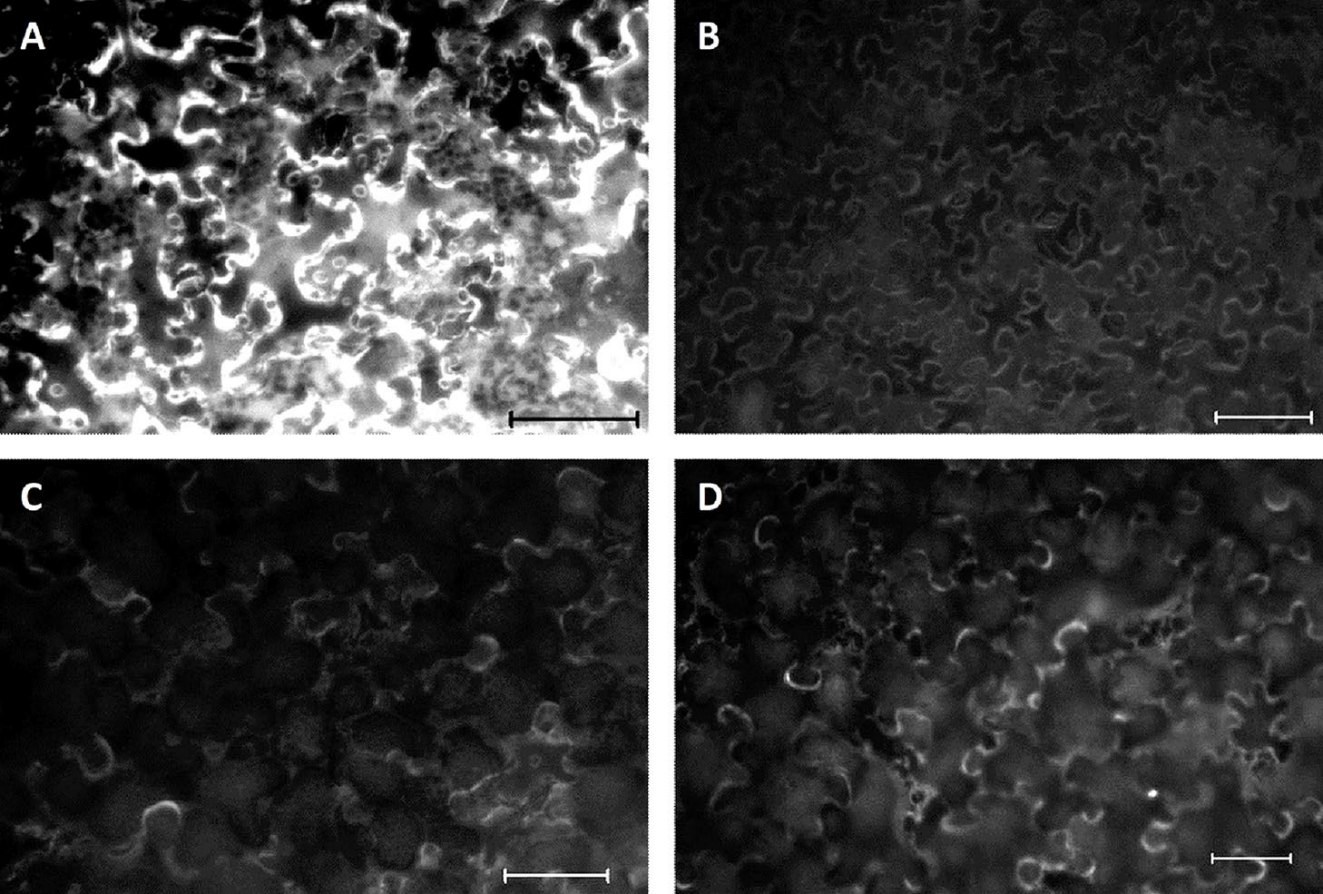
Fluorescent images of *Nicotiana benthamiana* leaf discs infiltrated with the over-expressing VvGGT3 construct five days prior to the addition of the γ-glutamyl-7-amido-4-methylcoumarin substrate. Images are representative of the result obtained from at least 10 independent infiltrations. **(A)** Leaves transiently transformed with the VvGGT3 over-expressing construct and subsequently infiltrated with the substrate. **(B)** Untransformed leaves at the same age infiltrated with the substrate. **(C)** Leaves transiently transformed with the VvGGT3 over-expressing construct and subsequently infiltrated with molecular grade water. **(D)** Leaves transiently transformed with *Agrobacterium tumefaciens* GV3101 (MP90) without a transgene and infiltrated with the substrate. Scale bars = 100 μm.

Determination of the biochemical function of VvGGT3 using recombinant protein from over-expression experiments in *E. coli* was not successful due to difficulties in obtaining soluble protein. Similar difficulties have also been previously reported (Kobayashi et al., 2011), and have been partly attributed to the propensity for the recombinant protein to remain insoluble and self-cleave (Suzuki and Kumagai, 2002; Kobayashi et al., 2011). We, therefore, opted to determine whether VvGGT3 encoded an active GGT by using a plant-specific recombinant protein vector, pEAQ-HT-DEST2, (Sainsbury et al., 2009; Peyret and Lomonossoff, 2013) by transient over-expression of the VvGGT3 open reading frame in *N. benthamiana* leaves. Attempts to purify the resulting 6xHis tagged protein from the leaves also proved difficult. Finally, we decided to determine if functional recombinant VvGGT3 protein was being produced in the transiently transformed *N. benthamiana* leaves by infiltrating a quenched GGT fluorescent substrate (γ-glutamyl-7-amido-4-methylcoumarin) and directly observing the enzymatic release of fluorescent 7-amido-4-methylcoumarin (**Figure 2A**). Controls were included to confirm that the observed fluorescence was due to VvGGT3-induced cleavage and not due to either auto-degradation of the substrate, or due to autofluorescence, or due to the infiltration of *Agrobacterium* GV3101 (MP90) resulting in pathogen-induced expression of endogenous *N. benthamiana* GGTs. These consisted of untransformed leaves infiltrated with γ-glutamyl-7-amido-4-methylcoumarin (**Figure 2B**), VvGGT3 transformed leaves, which were subsequently infiltrated with molecular grade water (**Figure 2C**) and leaves infiltrated with untransformed *Agrobacterium* GV3101 (MP90) and subsequently infiltrated with the substrate (**Figure 2D**), respectively. In each case, the controls did not show the same accumulation of fluorescence as seen in **Figure 2A**. These results demonstrates that *VvGGT3* encodes a functional GGT when transiently over-expressed in *N. benthamiana* leaves and that the location of the enzymatic activity did not appear to localize to the vacuole as predicted based on sequence similarity, but rather in an undetermined location associated with cellular membrane structures.

**Figure 3:**
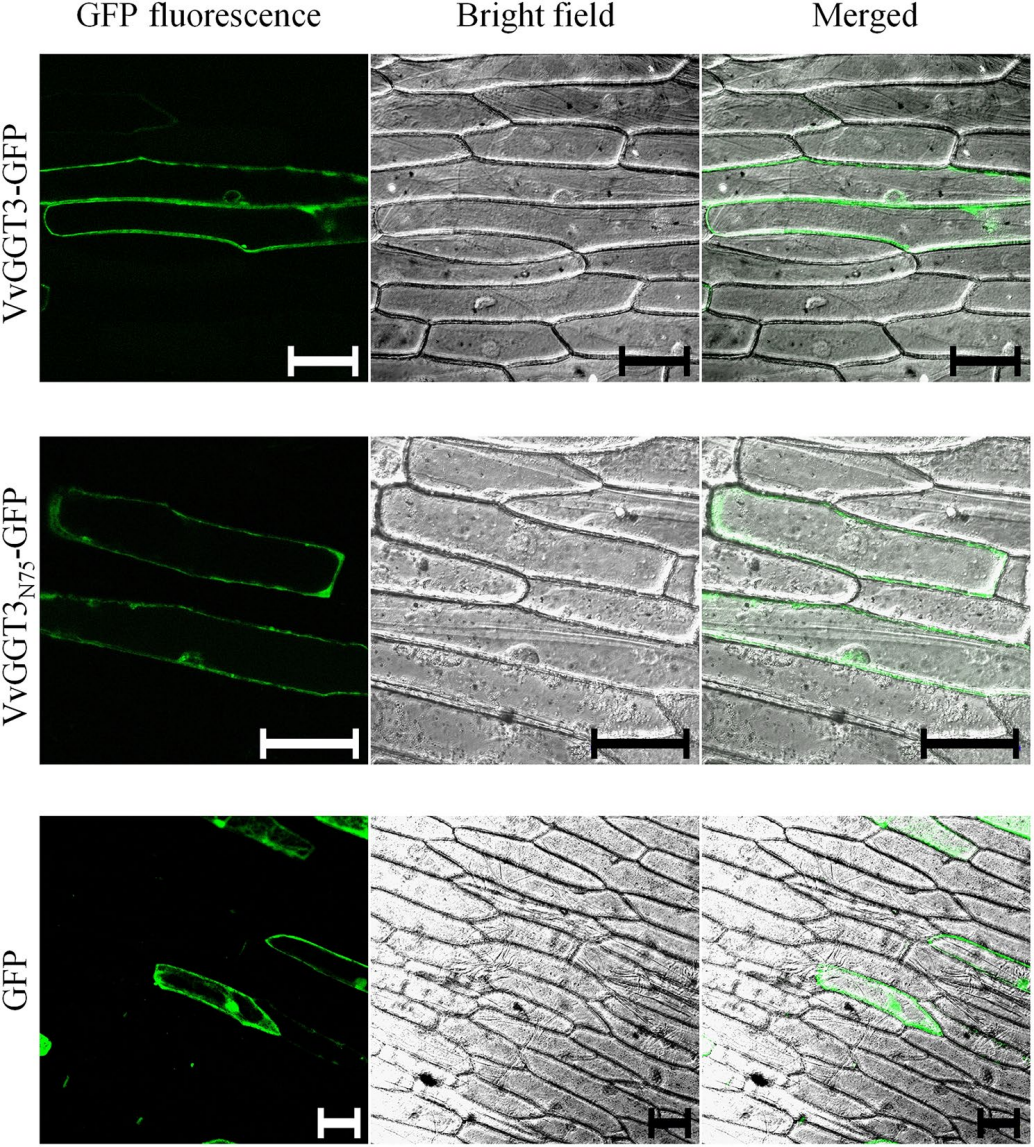
Confocal images of onion cells transiently transformed with either: the VvGGT3-GFP fusion protein **(top row)**, VvGGT3_N75_-GFP fusion protein **(middle row)**, or GFP alone **(bottom row)**. The merged images demonstrate that neither the full length (VvGGT3) nor the first 75 AA of the protein (VvGGT3_N75_) localize to the vacuole as predicted by phylogenetic analysis. The free GFP localizes to the nucleus and cell membranes as described in Ohkama-Ohtsu et al. (2007b). The images presented are representative of cells obtained from epidermal peels from three independent biolistic events on three individual bulb leaf sections bulb for each construct. Each peel was inspected to compare cells from at least three different fields of view. Two independent experiments carried out. Scale bars = 100 µm.

To further resolve the subcellular localization of VvGGT3, we transiently transformed onion epidermal cells with a VvGGT3-GFP full-length translational fusion construct. The VvGGT3-GFP fusion protein was found to be associated with what appears to be membrane structures or the tonoplast (**Figure 3**, top row). A 3D rendered video of the z-stacked confocal image captured in the top row of (**Figure 3**) demonstrates that the VvGGT3-GFP fusion protein does not localize to the vacuole (**Supplementary Video 1**). The pattern of accumulation is also consistent with that observed for the *in vivo* enzymatic activity (**Figure 2**), indicating that the site of enzyme activity is not within the vacuole as predicted by VvGGT3 subgrouping with AtGGT4 and AsGGT2.

In garlic, weak fluorescent signals have been detected when full-length AsGGTs or the first 300 AA residues have been fused to GFP and the authors suggest that this may be in part due to the incorrect processing of the AsGGT-GFP fusion (Yoshimoto et al., 2015). A stronger fluorescent signal was then produced when the authors instead used the first 100 AA from AsGGTs for their localization experiments (Yoshimoto et al., 2015). To test if the full-length VvGGT3-GFP fusion was unstable or incorrectly processed, we fused the first 75 AA of VvGGT3 (VvGGT3_N75_) to GFP and found the same localization patterns (**Figure 3**, middle row). We then transiently transformed onion epidermal cells with free GFP. Free GFP was shown to localize to the nucleus and cell membranes, as demonstrated in Ohkama-Ohtsu et al. (2007b), which is different to that observed by the VvGGT-GFP fusion constructs. We also captured similar localization patterns upon transiently transformation in *N. benthamiana* leaf cells (**Supplementary Figure 4**). In addition, the fluorescence of the VvGGT3-GFP fusion constructs are in accordance with the computational programs WoLF PSORT (Horton et al., 2007) and PSORT (Nakai and Horton, 1999), both predicting VvGGT3 to be localized to membranous compartments.

### *VvGGT3* Transcript Accumulation Profiles in Grapevine

There has been the suggestion that plant GGTs are regulated in response to stress, such as oxidative stresses in *Arabidopsis*, (Ohkama-Ohtsu et al., 2009), γ-irradiation to garlic bulbs after harvest (Ceci et al., 1992), and UV-C and temperature exposure to grape berries (Kobayashi et al., 2011). With only a single GGT isoform identified in grapevine and considering the numerous roles that GGTs are proposed to play within plants, we were interested to determine the patterns of *VvGGT3* transcript accumulation. In particular, due to the proposed roles of VvGGT3 in the formation of important flavor and aroma precursors in grape berries, we were very interested to discover the patterns of transcript accumulation between berry fractions, across berry development, and in response to exposure of tissues to biotic and abiotic stress treatments.

**Figure 4:**
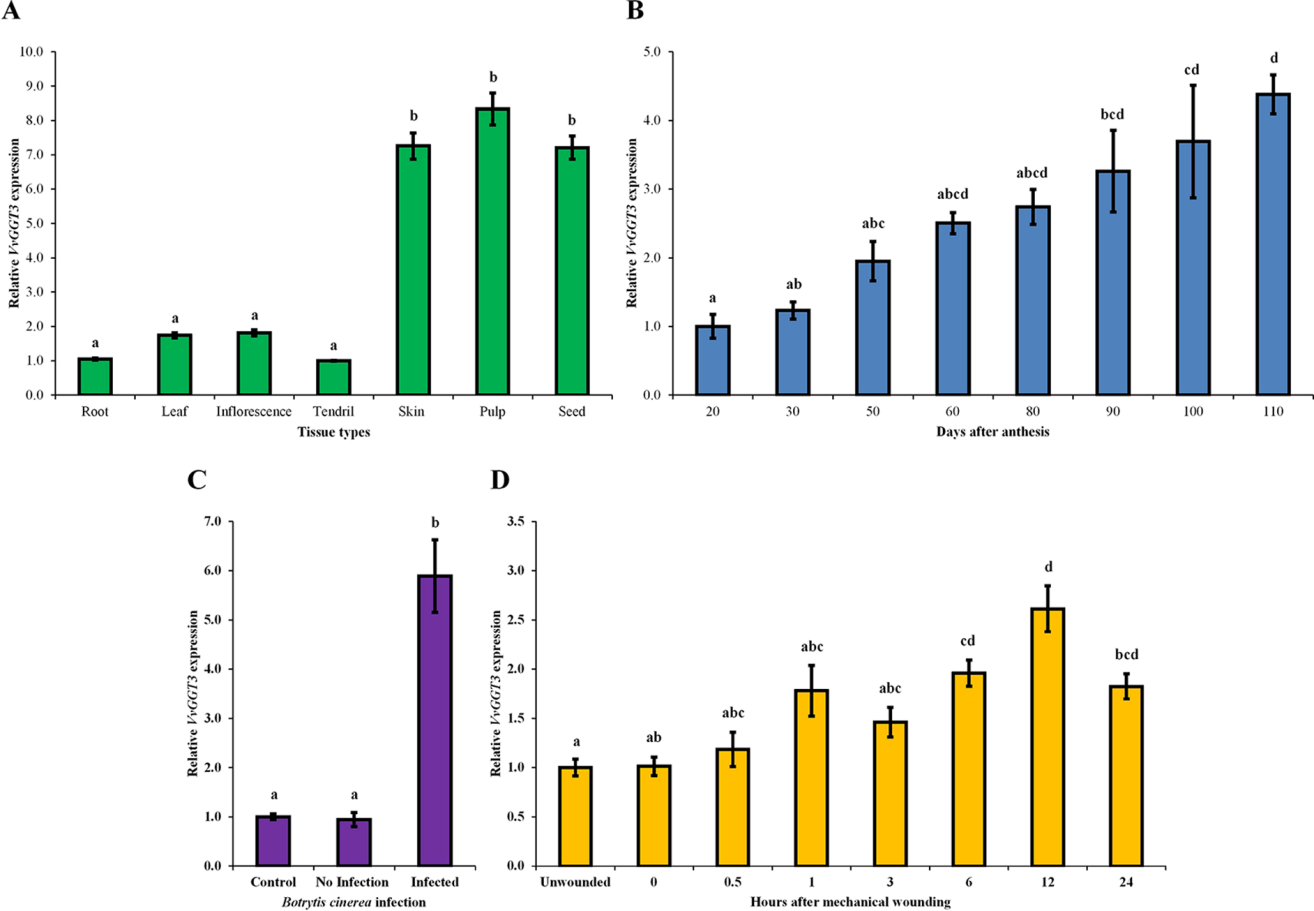
The mean relative abundance of *VvGGT3* in a range of Sauvignon blanc tissues and stress treatments. All data is derived from biological replicates and technical triplicates were performed for each RT-qPCR gene targets. **(A)** The mean relative abundance in root (root tip, ∼15 mm), leaf (∼225 mm^2^), E-L 15 inflorescence (Coombe, 1995), and tendril (∼20 mm), and the skin, pulp, and seed fraction of mature berries [21°Brix, mostly E-L 38 berries (Coombe, 1995)], the expression level of tendril was set to 1. **(B)** The mean relative abundance in grape berries throughout development was studied in the 2006, 2007, 2008, and 2009 growing seasons. For each time point, the phenological stages mostly represented E-L 30, 31, 32, 35, 36, 36, 37, and 38, respectively (Coombe, 1995). Veraison in each season took place approximately 50 to 60 days after anthesis (daa). The level at 20 daa was set to 1 (n = 4 biological replicates, n = 3 biological replicates for 20 and 30 daa). **(C)** The mean relative abundance in whole grape berries from bunches prior to harvest [21 °Brix, mostly E-L 38 berries (Coombe, 1995)] infected with *B. cinerea*. Berries that showed no sign of infection were considered the control and set to 1, berries of healthy appearance adjacent to infected berries were considered not infected (no infection), and grape berries that showed signs of infection but were not fully infected (infected) were analyzed (**Supplementary Figure 6**). **(D)** The mean relative abundance over 24 h in mechanically damaged young grape leaves (∼2.5–3 cm wide). The level of unwounded leaves was set to 1. For all assays (A–D), the geometrical means of *VvActin* and *VvEF1α* as reference genes were used as a normalization factors. Different letters indicate statistically significant differences, *P* < 0.05, ANOVA Tukey-Kramer, n = 3 biological replicates for graphs in (A), (C), and (D), means ± SEM.

The *VvGGT3* transcript was found to be expressed in all tissues tested (roots, leaf, inflorescences, and tendril, and in the skin, pulp, and seed fractions of mature grape berries (**Figure 4A**). The transcript abundance of *VvGGT3* was the lowest in the tendril, followed by the root, with a 1.7- and 1.8-fold increase in leaf and inflorescence compared to the tendril, respectively (**Figure 4A**). Focusing on the relative *VvGGT3* abundance in the skin, pulp, and seed fractions of mature grape berries, we observed similar levels of transcript in all three berry tissues, with these all being over 7-fold higher than the tendril (**Figure 4A**).

Across whole berry development, *VvGGT3* accumulated continuously, peaking at harvest (110 days after anthesis—berries between 21 and 23 ˚Brix; **Figure 4B**). Across the individual seasons, we saw a relatively consistent pattern of accumulation, indicating that while there are clearly some inter-seasonal variations, *VvGGT3* transcript accumulation is largely under developmental regulation (**Supplementary Figure 5**).

VvGGT3 has a proposed role in the formation of precursors of key varietal aromas in wine (Kobayashi et al., 2011). These compounds are considered to be derived from the lipoxygenase/hydroperoxide lyase mediated breakdown of linolenic and linoleic C18 fatty acids to produce C6 hexanal and then its derivatives (Capone and Jeffery, 2011; Kobayashi et al., 2011; Thibon et al., 2011; Capone et al., 2012). These derivatives are then thought to be conjugated to GSH by GSTs and are potential substrates for GGT activity within the vacuole of the berry. Levels of C6-GSH, C6-Cys-Gly, and C6-Cys accumulation in grape berries and juice have been shown to increase when the berries have been subjected to pathogens such as *B. cinerea* and/or wound stress (Capone and Jeffery, 2011; Kobayashi et al., 2011; Thibon et al., 2011). Therefore, we investigated whether either of these stresses might also impact *VvGGT3* gene transcription in Sauvignon blanc. To test the impact of *Botrytis* infection on *VvGGT3* transcript accumulation, we isolated berries from infected bunches that had (i) no sign of infection, (ii) berries on infected bunches immediately adjacent to infected berries but did not show sign of infection, and (iii) berries that showed signs of *Botrytis* infection, but were not fully infected (**Supplementary Figure 6**). As expected, **Figure 4C** clearly shows a correlation between *Botrytis* infection and a strong 5.8-fold upregulation of the *VvGGT3* transcript. To test the impact of wounding on *VvGGT3* transcription, we mechanically wounded leaves and collected leaf samples at regular intervals up to 24 h. In comparison to *Botrytis* infection, there is only a mild (∼2.0-fold) increase in the relative transcript abundance of *VvGGT3* compared to the unwounded control leaves at 6 h post wounding. This mild increase remains apparent at 12 h (∼2.6-fold) and at 24 h (∼1.8-fold), which are statistically different (*P* < 0.05) to that of the unwounded control leaves (**Figure 4D**).

## Discussion

### *V. vinifera* Possesses Only a Single GGT Gene

Analysis of the 12X.v2 *V. vinifera* reference genome revealed three potential GGT isoforms, with one isoform having three splice variants. After aligning these potential isoforms to functionally characterized GGTs from a range of species, candidates VIT_212s0142g00530 and VIT_201s0146g00200 do not possess conserved residues (Okada et al., 2006; Taniguchi and Ikeda, 2006) or the GGT molecular signature, [T-[STA]-H-x-[ST]-[LIVMA]-x(4)-G-[SN]-x-V-[STA]-x-T-x-T-[LIVM]-[NE]-x(1,2)-[FY]-G] (Ferretti et al., 2009) that are characteristic of GGT enzymes (**Supplementary Figure 3**). In addition, splice variant VIT_211s0016g02830.2, is missing the first conserved residue, R107 (in human) needed of GGT substrate binding (Taniguchi and Ikeda, 2006). Whereas, splice variant VIT_211s0016g02830.3 does not have conserved residues at its C-terminus, when compared to the selection of other plant GGT sequences (**Supplementary Figure 3**). It is therefore likely that these splice variants are mis-annotated. The remaining splice variant VIT_211s0016g02830.1, is identical in length to the reference XP_002280190.1. We isolated this GGT isoform from Sauvignon blanc and also found it to be the same length, we also find SNPs that are distinctive to this cultivar and have deposited this sequence to NCBI (MN101215.3). Thus, we only find a single GGT isoform in *V. vinifera*, which is in contrast to most other plants where two or more GGT isoforms exist (**Table 1**), tending to group into two distinct subgroups. Based on the characterized GGTs from *Arabidopsis* and garlic, these subgroups could potentially be GGTs that are either targeted to the vacuole or apoplast (**Figure 1**).

Given the range of roles that GGT enzymes are proposed to play in the maintenance of cellular activities, possession of only a single isoform in grapevine is intriguing. Phylogenetic comparison of the VvGGT3 putative peptide sequences with other plant GGT sequences shows that VvGGT3 associates in the subgroup containing the characterized AtGGT4 and AsGGT2, both of which localize to vacuoles and have presumed roles in the catabolism of GSH conjugated to a wide range of small molecules (Grzam et al., 2007; Yoshimoto et al., 2015). This is consistent with the proposed role of VvGGT3 in the formation of precursors of volatile thiols (e.g. C6-Cys-Gly, C6-Cys). However, it also raises questions as to how other roles proposed for GGTs in grapevine, in particular, maintenance of cellular redox potential *via* modulation of cellular GSH levels, are achieved? It is possible that an alternative GSH degradation pathway exists that is present in the cytoplasm rather than the apoplast or vacuole which utilizes γ-glutamyl cyclotransferases (GGCT) and 5-oxoprolinase (5OPase) rather than GGT (Ohkama-Ohtsu et al., 2008). As the cleavage of the γ-glutamyl bond is only known to be carried out by GGT, our results suggest that the range of activities proposed for GGT in grapevine is carried out by this single VvGGT3 isoform.

### Localization of VvGGT3

Due to difficulties in generating stable transgenic lines in *V. vinifera* (Sauvignon blanc), we were unable to investigate the impact of a *Vvggt3* knockout in grape. Therefore, we opted to study whether recombinant VvGGT3 possessed GGT activity by transient over-expression in *N. benthamiana* leaves and then supplying the leaf sections the quenched substrate γ-glutamyl-7-amido-4-methylcoumarin. As determined by the GGT-mediated cleavage of the γ-glutamyl moiety and release of fluorescent 7-amido-4-methylcoumarin, VvGGT3 is indeed functional and the substrate does not accumulate in the vacuole (**Figure 2A**).

To investigate the localization of VvGGT3, we generated a genetic construct designed to produce a C-terminal translational fusion of GFP with the full-length VvGGT3 protein. When this fusion protein construct was transiently transformed into onion epidermal cells (**Figure 3**, **Supplementary Video 1**) and *N. benthamiana* leaves (**Supplementary Figure 4**), fluorescence of the VvGGT3-GFP fusion protein was not detected in the vacuole, but was rather associated with membranes. This result complements the location of the GGT-cleaved 7-amido-4-methylcoumarin substrate seen in **Figure 2A**. Close inspection of the GFP images indicates that the localization may be in the cytoplasm or also associated with the tonoplast but is clearly not in the vacuole.

In garlic, weak fluorescent signals were detected when the full-length AsGGTs or the first 300 AA residues were used in GFP-fusion constructs and the authors suggest that this may be in part due to the incorrect processing of the AsGGT-GFP fusion (Yoshimoto et al., 2015). Similarly, in *Arabidopsis*, no fluorescence was detected when the full-length AtGGT4 or the first 300 AA residues were used in GFP-fusion constructs (Ohkama-Ohtsu et al., 2007b). Fluorescence was detected when the first 100 AA were instead used in localization experiments (Ohkama-Ohtsu et al., 2007b; Yoshimoto et al., 2015). To determine whether the full-length VvGGT3 sequence was unstable or incorrectly processed, we fused the first 75 amino acids of VvGGT3 (VvGGT3_N75_) to GFP and found the same localization patterns. This result indicates that there was no difference in the stability or processing of either the full length (VvGGT3-GFP) or truncated (VvGGT3_N75_-GFP) fusions (**Figure 3**).

The apoplast generally has an acidic pH (<6.5) and GFP fusion proteins have been reported to show poor to variable fluorescence in this compartment (Schulte et al., 2006). This is due to reversible protonation of GFP leading to quenching of fluorescence when subjected to a pH range between pH 7 and 5 (Schulte et al., 2006). If the pH drops below pH 5, then an irreversible conformational change of GFP takes place leading to protein instability (Schulte et al., 2006). Thus, the strong fluorescence we have observed with the transient expression of the VvGGT3-GFP fusion in onion epidermal cells argues against an apoplastic location for the fusion protein. However, further experiments are needed to conclusively determine the subcellular localization of VvGGT3 and the apoplast cannot be dismissed.

Despite the bioinformatic prediction of the vacuolar localization for VvGGT3, the localization of the VvGGT3-GFP fusion protein as well as the corresponding enzymatic activity in *N. benthamiana* suggest that VvGGT3 is unlikely to be involved in the processing of GSH-conjugated metabolites in the vacuoles of grapevine cells. In addition, due to the strong GFP signal of the VvGGT3-GFP fusion protein, the enzyme is also unlikely to be involved in the apoplastic recycling of GSSH. The presumed absence of a GGT in grape vacuoles also raises important questions as to the subcellular localization of enzymatic activities responsible for green leaf volatile and green leaf volatile conjugate formation in grapevine. In particular, these data raise questions as to the possible mechanisms for the production of the Cys-Gly and Cys-precursors of the volatile C6-thiolated metabolites that are important for Sauvignon blanc flavors and aromas. There is little data on the physical storage location of these precursors in grapevine and data suggest that it may be possible that the removal of the γ-Glu from GSSH conjugated metabolites may occur (leaving a Cys-Gly conjugate) in the cytoplasm prior to transport of these conjugated metabolites into the vacuole. There are alternate recycling pathways for GSSH reported in plants that involve GGCT and 5OPase (Ohkama-Ohtsu et al., 2008). In *Arabidopsis*, this recycling pathway has been shown to be the main metabolic flux route for recycling of GSSH compared to the GGT route (Ohkama-Ohtsu et al., 2008). Thus, there is a possibility that the metabolism of both apoplastic GSSH and GSSH conjugates in the vacuole may preferentially use this alternative pathway in grapevine. At this time we cannot rule out these results are an artifact of the transient expression of these gene constructs in a heterologous species and consequently further work in grapevine would be required to determine the validity of this data.

### *VvGGT3* Transcript Abundance

Our main interest in VvGGT3 is its potential role in the formation of important precursors of the volatile thiol aromas (3MH and 3MHA) in grape berries that are characteristic of Sauvignon blanc wines. Consequently, we explored the pattern of *VvGGT3* transcript accumulation across tissues and with a focus on grape berries. It is clear that *VvGGT3* is expressed in every tissue tested (**Figure 4**) and thus, a sole GGT in grape is consistent with a presumed wide range of roles GGTs play in cellular metabolism, regulation, and recovery of GSH as well as acting on glutathionylated xenobiotics. Exploration of the patterns of transcript accumulation in mature berry tissues revealed a fairly even distribution of transcript abundance between the skin, pulp, and seed fractions (**Figure 4A**). However, the levels of *VvGGT3* transcript seen to be accumulating in mature berry pulp is surprising given the debate as to the varying levels of cell viability recorded for the berry pulp across a range of grape varietals (Tilbrook and Tyerman, 2008; Fuentes et al., 2010), which would otherwise result in global transcript reduction. Therefore, while we are able to detect significant levels of *VvGGT3* transcript in the total RNA isolated from mature berry pulp, we are not able to comment on the levels of active protein in these tissues. GGTs are known to accumulate in certain storage tissues such as seed and bulbs (Balakrishna and Prabhune, 2014; Sun et al., 2019). When stored at cool temperatures below 20°C, GGT activity has been detected in the bulbs of garlic and onion. This activity increases over time and can be detected up to eight months after storage (Hanum et al., 1995; Li et al., 2008). Similarly, in *Arabidopsis* seed, amongst the AtGGT members, AtGGT4, which based on sequence alignment is the closest match to VvGGT3, has the highest expression (Martin et al., 2007). Thus, taken together, it is not surprising that the expression of *VvGGT3* in a storage tissue such as the seed is high when compared to the other tissues tested.

In tomato fruit (cv. Alisa Craig and Rutgers), GGT activity was shown to increase during ripening, reviewed in Sorrequieta et al. (2010). Similarly, across whole berry development, *VvGGT3* demonstrates a general pattern of increasing in transcript abundance (**Figure 4B**). Grape berries feature a distinctive stage during the development known as veraison, where significant changes at a physiological level take place. These changes include, softening of the berry, accumulation of sugars, reduction in acids and changes in color in red varieties that signal the onset of ripening. Veraison is also associated with an oxidative burst and changes in cell structure, where cell division stops and berry enlargement occurs, mainly due to cell expansion (Coombe, 1992; Podolyan et al., 2010). Such physiological changes at veraison (50–60 days after anthesis) could account for the 2- to 2.5-fold increase in transcript abundance of *VvGGT3* (**Figure 4B**). In considering the accumulation of the *VvGGT3* transcript across four seasons, we clearly notice some inter-seasonal variation at each time point, which are likely to be due to differing climates (**Supplementary Figure 5**). However, the pattern of accumulation is generally stable, indicating that the main drivers for *VvGGT3* transcription are related to berry development and ripening. This pattern of accumulation is similar to patterns of transcript abundance for other genes involved in volatile thiol precursor formation [namely *VvLOX*, *VvHPL*, and *VvGST* (Podolyan et al., 2010; Kobayashi et al., 2011; Zhu et al., 2012)].

In the context of flavors and aroma, wine produced from grapes infected by *B. cinerea* have a considerably higher concentration of volatile thiols (especially 3MH), reviewed by Coetzee and du Toit (2012). This is due in part to the berry's increase in the precursors such as 3MH-*S*-Cys (Thibon et al., 2009). This precursor is most likely formed after GGT and carboxypeptidase enzymes have cleaved Glu and Gly from the GSH-conjugate. Evidence of the role GGT plays in this formation was demonstrated by des Gachons et al. (2002), who showed 3MH-*S*-Cys to be liberated from 3MH-*S*-glut in grape must after passing the must through a 4B sepharose column with immobilized copper (II) and the GGT enzyme.

*B. cinerea* is not found to be directly responsible for this precursor formation but most likely stimulates the grape metabolic pathway involved in its formation due to the release of the fungal mebabolites (Thibon et al., 2011). In stressed plants, the lipoxygenase-hydroperoxide lyase pathway releases C6 volatiles and other reactive species, reviewed by Bate and Rothstein (1998); Thibon et al. (2011). More specifically, in grape berries, the lipoxygenase, *VvLOXC* and *VvLOXO*, transcripts are upregulated when infected by *B. cinerea* (Podolyan et al., 2010), this in turn may induce the detoxification pathway as the C6 compounds produced are not only toxic to the pathogen but at a critical level can become toxic to the plant itself (Coetzee and du Toit, 2012). Thus, to decrease toxicity, these compounds can be conjugated to GSH *via* the GST enzymes. In grape berry skins, the transcript of *VvGST1*, *VvGST3*, and *VvGST4* are found to be upregulated when infected with the downy mildew pathogen, which in turn leads to an increase in the concentrations of GSH-conjugates (Kobayashi et al., 2011). In addition, the *VvGGT3* transcript was upregulated 1.7-fold (Kobayashi et al., 2011) and is likely to be involved in the breakdown of the GSH-conjugates, which include the flavor and aroma precursor compounds as demonstrated by a higher production of the 3MH-*S*-Cys precursor (Kobayashi et al., 2011). Similarly, in our study, while a general pattern of developmentally regulated *VvGGT3* transcript accumulation is evident, we also show a strong 5.8-fold pathogen related increase for *VvGGT3* in mature grape berries (**Figure 4C**). However, we see only small levels of transcript accumulation in response to wounding of young grape leaves. Collectively, these data point to an important role for pathogen infection rather than wounding in modulating the levels of these important flavor and aroma precursors in grapevine.

## Conclusion

Based on a bioinformatics search in *V. vinifera*, we have functionally characterized a sole GGT enzyme, VvGGT3. Our substrate localization and VvGGT3-GFP fusion results indicate that VvGGT3 does not localize to the vacuole as predicted by phylogenetic analysis. Rather, it is localized to membrane structures. This raises important questions about the subcellular localization for production transport and storage of C6 volatiles and their conjugates in grapevine. The *VvGGT3* transcript was detected in all tissue tested and was most abundant in the skin, pulp, and seed fractions of mature berries. In whole berries, *VvGGT3* appears to be under developmental control as the transcript steadily increases over time. We observed *VvGGT3* transcript upregulation in whole berries infected with *B. cinerea* and in mechanically wounded leaves, which is not surprising given the production of GGT-cleavable volatiles upon biotic and abiotic stresses. The sole GGT member in grapevine is intriguing given the range of roles GGTs play *in planta*, the findings presented here may be foundational for other crop species where only a single GGT may exist.

## Data Availability Statement

The VvGGT3 sequence from Sauvignon blanc can be found in GenBank accession MN101215.3.

## Author Contributions

JP and CW designed the experiments. JP performed experiments and analyzed all data. WD performed leaf wounding and tissue type qRT-PCR experiments. CW performed GFP localization experiments in onion cells. CW supervised the experiments. JP and CW wrote the original draft. JP and CW reviewed and edited the manuscript.

## Funding

This work was supported by Lincoln University and the Ministry of Business, Innovation and Employment (previously the New Zealand Foundation for Research, Science and Technology), contract number UOAX0404.

## Conflict of Interest

The authors declare that the research was conducted in the absence of any commercial or financial relationships that could be construed as a potential conflict of interest.
