# Supplementary material for: Functional Characterization of the Grapevine γ-Glutamyl Transferase/Transpeptidase (E.C. 2.3.2.2) Gene Family Reveals a Single Functional Gene Whose Encoded Protein Product Is Not Located in Either the Vacuole or Apoplast
Source: Front Plant Sci. 2019 Nov 4;10:1402. doi: 10.3389/fpls.2019.01402 (PMC6843540; doi:10.3389/fpls.2019.01402)
Supplement: Supplementary file 1 [file DataSheet_1.pdf]

Supplementary Material

Functional Characterization of the Grapevine γ-Glutamyl Transferase/Transpeptidase (E.C. 2.3.2.2) Gene Family Reveals a Single Functional Gene Whose Encoded Protein Product Is Not Located in Either the Vacuole or Apoplast

Joshua G. Philips, Walftor Dumin & Christopher Winefield*

*** Correspondence:** Christopher Winefield: Christopher.winefield@lincoln.ac.nz

1 Supplementary Tables and Figures

1.1 Supplementary Tables

Supplementary Table S1. Primer sequences.

1.2 Supplementary Figures

Supplementary Figure S1. Phylogeny of selected γ-glutamyl transferases/transpeptidases (GGTs) from planta after removal of the N-terminal leader and targeting sequences. This was performed after multiple protein sequence alignments and exclusion of all residues prior to Val77 of VvGGT3, which is the first largely conserved (97.7%) residue. The accession numbers of these GGTs are presented in Table 1. GGTs group into two distinctive subgroups, then further into the monocot and dicot clades. Subgroup 1 has GGTs which are thought to prevent oxidative stress by degrading the oxidised form of glutathione (GSSG) and the breakdown of extracellular glutathione (GSH) in the apoplast. Subgroup 2 has GGTs which are thought to degrade GSH-conjugates in the vacuole. The sole GGT from grape, VvGGT3, highlighted in red, falls under subgroup 2. Multiple sequence alignments of deduced protein sequence that were used in this analysis can be found in Supplementary Data Sheet 3.

Supplementary Figure S2. Phylogeny of VvGGT isoforms and splice variants with selected γ-glutamyl transferases/transpeptidases (GGTs) from planta. The accession numbers of these GGTs are presented in Table 1. GGTs group into two distinctive subgroups, then further into the monocot and dicot clades. Subgroup 1 has GGTs which are thought to prevent oxidative stress by degrading the oxidised form of glutathione (GSSG) and the breakdown of extracellular glutathione (GSH) in the apoplast. Subgroup 2 has GGTs which are thought to degrade GSH-conjugates in the vacuole. All VvGGT candidates, along with VvGGT3, highlighted in red, fall under subgroup 2. The VIT_212s0142g00530 and VIT_201s0146g00200 candidates are the most divergent when compared to the other GGT sequences from planta. Multiple sequence alignments of deduced protein sequence that were used in this analysis can be found in Supplementary Data Sheet 4.

Supplementary Figure S3. (Previous page) Multiple sequence alignment of VvGGT isoforms and splice variants with selected, functionally characterised γ-glutamyl transferases/transpeptidases (GGTs) from planta, *E. coil*, human and rat. The accession numbers of these GGTs where not presented in Table 1 include: *E. coil* (AAA23869.1), human (P19440.2), rat (AAB59698.1), A0A438IHM7 accession from https://www.uniprot.org/, accessed 30/8/2019 and VIT accessions from http://genomes.cribi.unipd.it/grape/, accessed 21/8/2019. Key residues, essential for GGT activity are conserved in bacterial, mammalian and plant GGT sequences (red column). These residues include: R107 and D423 in human, which are involved in the binding of substrates (B) (Taniguchi and Ikeda, 2006). The catalytic nucleophile, T391 in *E. coli* (N), and the residue involved in stabilising the nucleophile, T409 (S) (Okada et al., 2006). The residues S451 and S452 in human, which are involved in enzyme catalysis (CC) (Okada et al., 2006; Taniguchi and Ikeda, 2006), and G483 and G484 in *E. coli*, which comprise the GGT oxyanion hole (OO) (Okada et al., 2006). The GGT molecular signature, [T-[STA]-H-x-[ST]-[LIVMA]-x(4)-G-[SN]-x-V-[STA]-x-T-x-T-[LIVM]-[NE]-x(1,2)-[FY]-G] (Ferretti et al., 2009) is shown by the blue box. The multiple sequence alignment was performed using the default ClustalW settings within Geneious 10.1.3 (Biomatters Ltd.), as described. Sequence identity shading; green - 100%, olive - 80-99%, yellow - 60-80% and white <60%, the alignment used to prepare this figure can be found in Supplementary Data Sheet 5.

Supplementary Figure S4. Confocal images of *Nicotiana benthamiana* leaf cells transiently transformed with either the VvGGT3-GFP fusion protein **(Top row),** VvGGT3$_{N75}$-GFP fusion protein **(Middle row)** or GFP alone **(Bottom row)**. The merged images demonstrate that neither the full length (VvGGT3) nor the first 75 amino acids (VvGGT3$_{N75}$) localise to the vacuole as predicted by phylogenetic analysis. The free GFP localises to the nucleus and cell membranes as described in Ohkama-Ohtsu et al., 2007b.

Supplementary Figure S5. The mean relative abundance of *VvGGT3* in grape berries throughout development was studied in the 2006, 2007, 2008 and 2009 growing seasons. For each time-point, the phenologcal stages mostly represented E-L 30, 31, 32, 35, 36, 36, 37 and 38, respectively (Coombe, 1995). Veraison in each season took place approximately 50 to 60 days after anthesis (daa). The mean level at 20 daa across all growing seasons was set to 1. Data not available at 20 daa in the 2006 growing season and excluded at 30 daa in the 2008 growing season (N/A). The geometrical means of *VvActin* and *VvEF1α* as reference genes were used as a normalisation factors, n=3 technical replicates, means ± SEM. The mean relative abundance from each season was used as biological replicates to prepare Figure 4B.

Supplementary Figure S6. Image of a *Botrytis cinerea* infected grape bunch. For *VvGGT3* transcript accumulation, we isolated berries from infected bunches that had (i) no sign of infection (Control), (ii) berries on infected bunches immediately adjacent to infected berries but did not show sign of infection (No Infection) and (iii) berries that showed signs of Botrytis infection, but were not fully infected (Infection).

1.3 Supplementary Data Sheets

File names for Supplementary Data Sheets.

Data Sheet 2.FASTA – GGT full protein alignment used to produce Figure 1

Data Sheet 3.FASTA – GGT N-terminus truncated protein alignment used to produce Supplementary Figure S1

Data Sheet 4.FASTA – GGT protein alignment with VvGGT isoforms and splice variants used to produce Supplementary Figure S2

Data Sheet 5.FASTA – GGT protein alignment with VvGGT isoforms and splice variants against *E. coil*, human and rat used to produce Supplementary Figure S3

Video 1.MP4 - A 3D rendered video of the z-stacked confocal image captured in the Top row of Figure 3 (VvGGT3-GFP)

Supplementary Table S1. Primer sequences.

Primer	Sequence 5' → 3'	Amplicon Size
VvGGT3 Gateway Forward	<u>CACC</u>TCATCTGGCAGTTGTCTATTT	1897 bp
VvGGT3 Reverse	TTAATCAAGAACTAAGGATCACACAGC	
VvGGT3 Reverse -STOP	CACAGCAGCTGGCCTCCCAT	
VvActin qPCR Forward	CTTGCATCCCTCAGCACCTT	82 bp
VvActin qPCR Reverse	TCCTGTGGACAATGGATGGA	
VvEF1α qPCR Forward	AAAATAAAGCGGACGATCTAT	85 bp
VvEF1α qPCR Reverse	GGAAGCCTCCTATCATCAAAA	
VvGGT3 qPCR Forward	TAGAAAGGGAGGGCATGCTGTTGA	91 bp
VvGGT3 qPCR Reverse	TCCACCTCCTATTCCACTTGCCAT	
VvGGT3 (N75) Gateway Forward	<u>CACC</u>ATGGGGCAGCCGGG	225 bp
VvGGT3 (N75) Reverse	ACCCTGCTTTGATTCAACGATGC	

Underlined sequence required for directional cloning into pENTR/D-TOPO

VvActin qPCR primers are as described in Reid et al. (2006)
VvEF1α qPCR primers are as described in Tashiro et al. (2016)

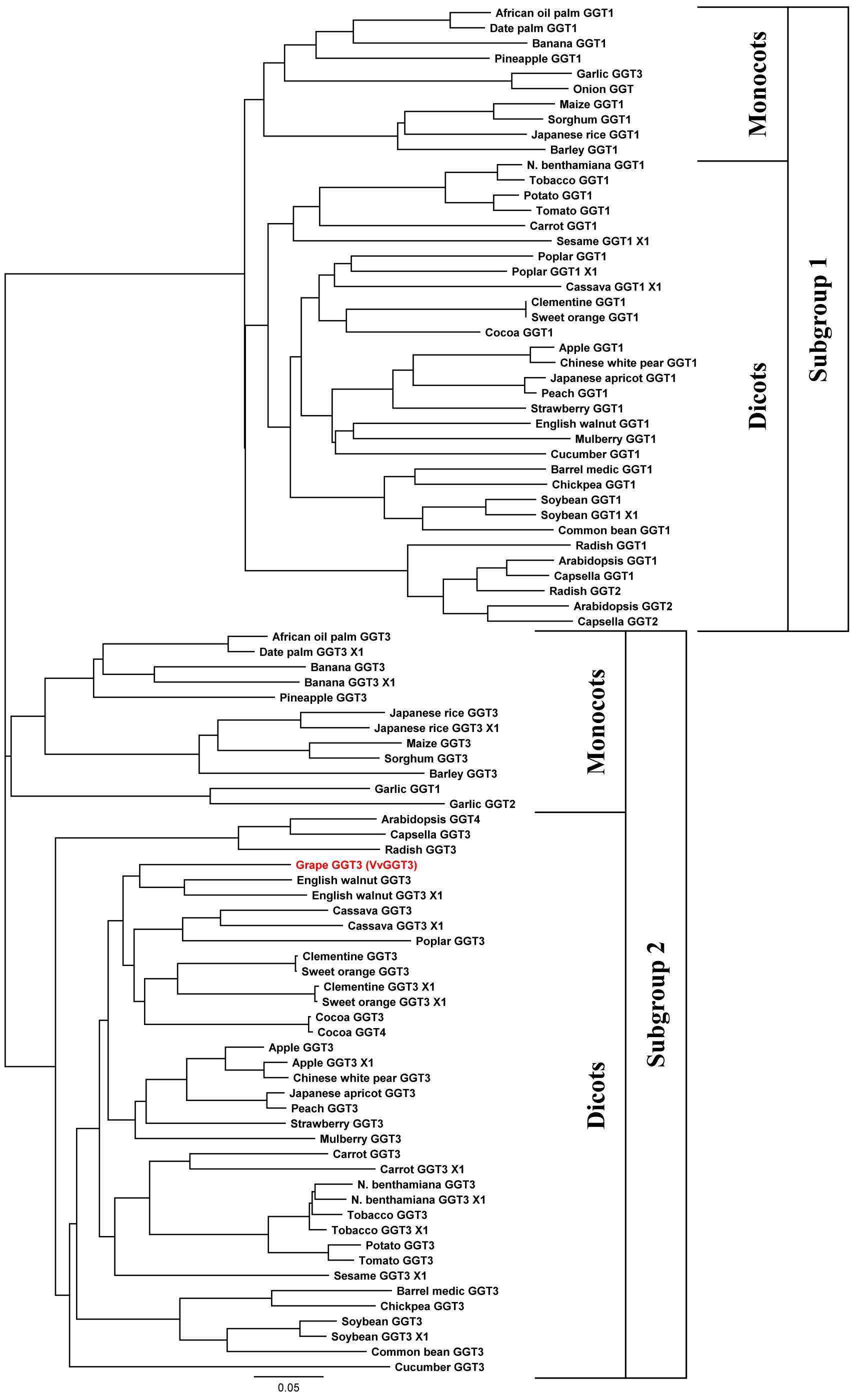

Supplementary Figure S1. Phylogeny of selected γ-glutamyl transferases/transpeptidases (GGTs) from planta after removal of the N-terminal leader and targeting sequences. This was performed after multiple protein sequence alignments and exclusion of all residues prior to Val77 of VvGGT3, which is the first largely conserved (97.7%) residue. The accession numbers of these GGTs are presented in Table 1. GGTs group into two distinctive subgroups, then further into the monocot and dicot clades. Subgroup 1 has GGTs which are thought to prevent oxidative stress by degrading the oxidised form of glutathione (GSSG) and the breakdown of extracellular glutathione (GSH) in the apoplast. Subgroup 2 has GGTs which are thought to degrade GSH-conjugates in the vacuole. The sole GGT from grape, VvGGT3, highlighted in red, falls under subgroup 2. Multiple sequence alignments of deduced protein sequence that were used in this analysis can be found in Supplementary Data Sheet 3.

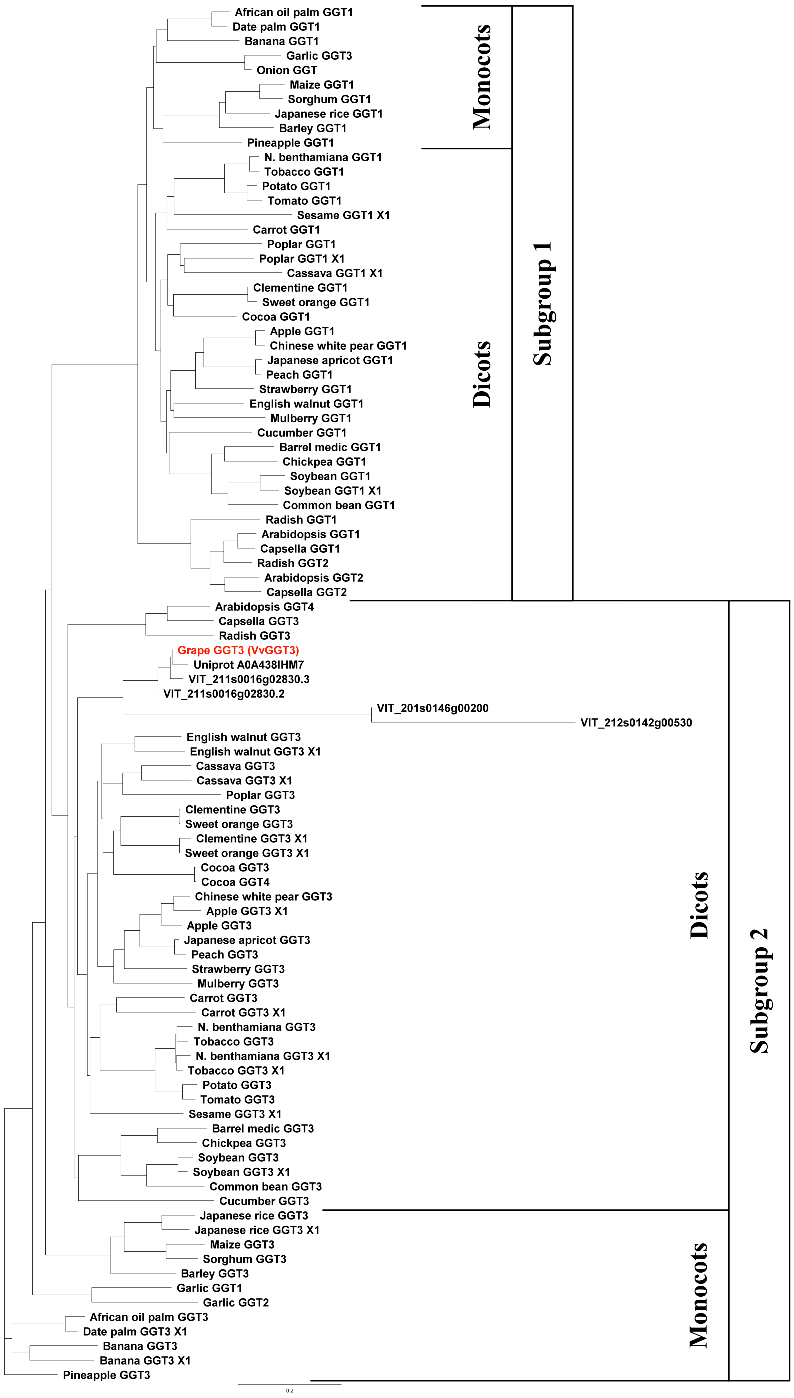

Supplementary Figure S2. Phylogeny of VvGGT isoforms and splice variants with selected γ-glutamyl transferases/transpeptidases (GGTs) from planta. The accession numbers of these GGTs are presented in Table 1. GGTs group into two distinctive subgroups, then further into the monocot and dicot clades. Subgroup 1 has GGTs which are thought to prevent oxidative stress by degrading the oxidised form of glutathione (GSSG) and the breakdown of extracellular glutathione (GSH) in the apoplast. Subgroup 2 has GGTs which are thought to degrade GSH-conjugates in the vacuole. All VvGGT candidates, along with VvGGT3, highlighted in red, fall under subgroup 2. The VIT_212s0142g00530 and VIT_201s0146g00200 candidates are the most divergent when compared to the other GGT sequences from planta. Multiple sequence alignments of deduced protein sequence that were used in this analysis can be found in Supplementary Data Sheet 4.

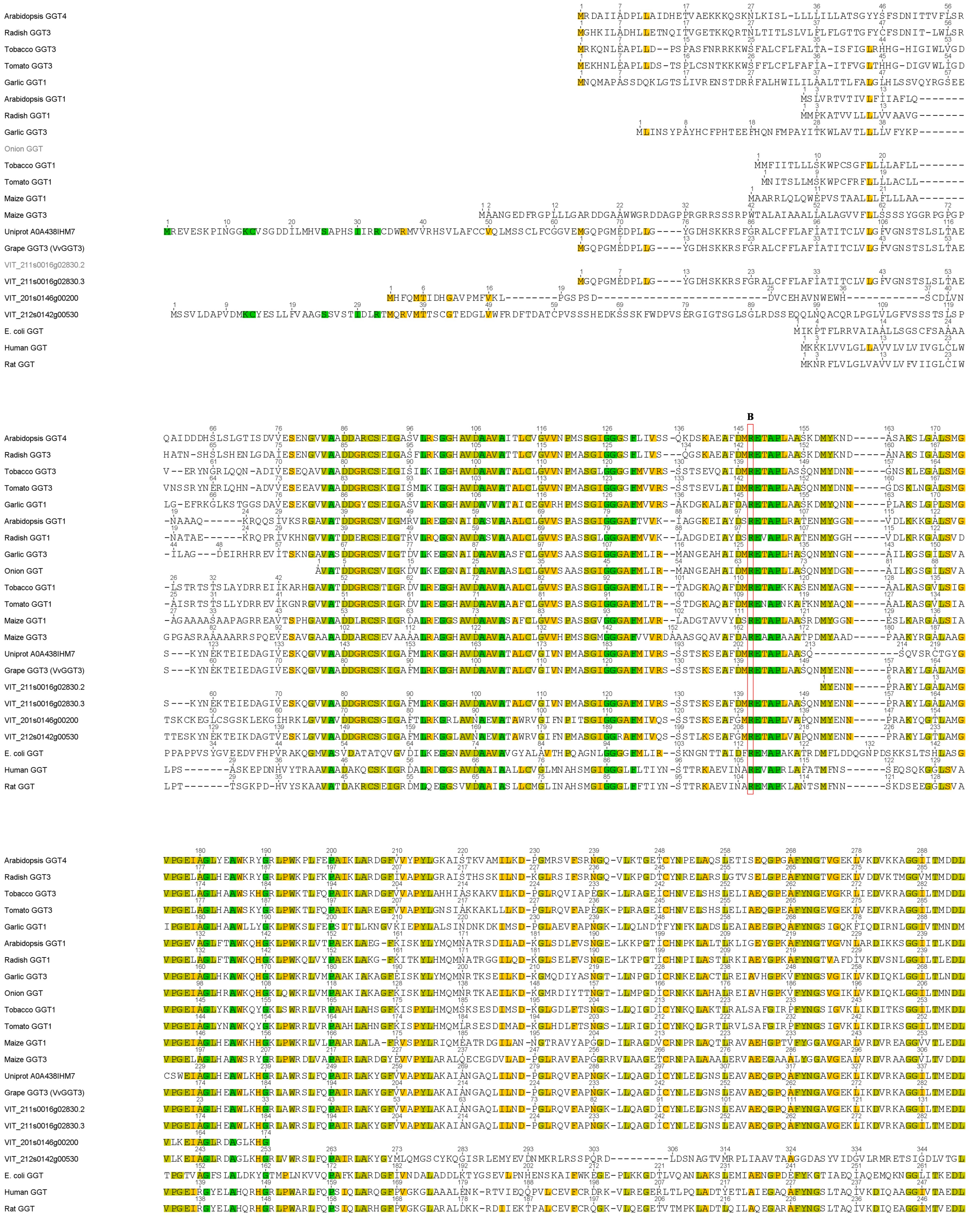

Arabidopsis GGT4
Radish GGT3
Tobacco GGT3
Tomato GGT3
Garlic GGT1
Arabidopsis GGT1
Radish GGT1
Garlic GGT3
Onion GGT
Tobacco GGT1
Tomato GGT1
Maize GGT1
Maize GGT3
Uniprot A0A438IHM7
Grape GGT3 (VvGGT3)
VIT_211s0016g02830.2
VIT_211s0016g02830.3
VIT_201s0146g00200
VIT_212s0142g00530
E. coli GGT
Human GGT
Rat GGT
B

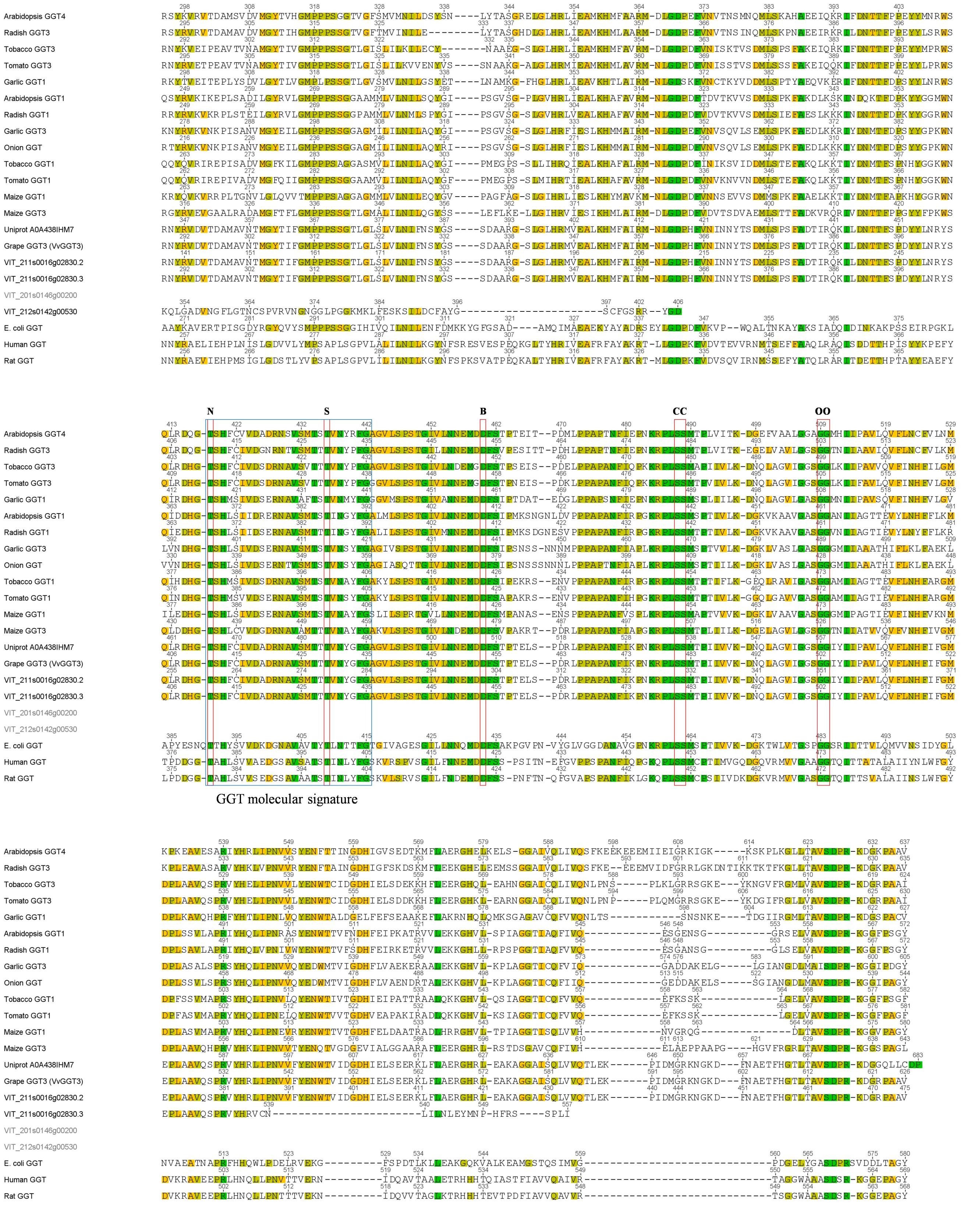
Arabidopsis GGT4
Radish GGT3
Tobacco GGT3
Tomato GGT3
Garlic GGT1
Arabidopsis GGT1
Radish GGT1
Garlic GGT3
Onion GGT
Tobacco GGT1
Tomato GGT1
Maize GGT1
Maize GGT3
Uniprot A0A438IHM7
Grape GGT3 (VvGGT3)
VIT_211s0016g02830.2
VIT_211s0016g02830.3
VIT_201s0146g00200
VIT_212s0142g00530
E. coli GGT
Human GGT
Rat GGT
N
S
B
CC
OO
GGT molecular signature

Supplementary Figure S3. (Previous page) Multiple sequence alignment of VvGGT isoforms and splice variants with selected, functionally characterised γ-glutamyl transferases/transpeptidases (GGTs) from planta, *E. coil*, human and rat. The accession numbers of these GGTs where not presented in Table 1 include: *E. coil* (AAA23869.1), human (P19440.2), rat (AAB59698.1), A0A438IHM7 accession from https://www.uniprot.org/, accessed 30/8/2019 and VIT accessions from http://genomes.cribi.unipd.it/grape/, accessed 21/8/2019. Key residues, essential for GGT activity are conserved in bacterial, mammalian and plant GGT sequences (red column). These residues include: R107 and D423 in human, which are involved in the binding of substrates (B) (Taniguchi and Ikeda, 2006). The catalytic nucleophile, T391 in *E. coli* (N), and the residue involved in stabilising the nucleophile, T409 (S) (Okada et al., 2006). The residues S451 and S452 in human, which are involved in enzyme catalysis (CC) (Okada et al., 2006; Taniguchi and Ikeda, 2006), and G483 and G484 in *E. coli*, which comprise the GGT oxyanion hole (OO) (Okada et al., 2006). The GGT molecular signature, [T-[STA]-H-x-[ST]-[LIVMA]-x(4)-G-[SN]-x-V-[STA]-x-T-x-T-[LIVM]-[NE]-x(1,2)-[FY]-G] (Ferretti et al., 2009) is shown by the blue box. The multiple sequence alignment was performed using the default ClustalW settings within Geneious 10.1.3 (Biomatters Ltd.), as described. Sequence identity shading; green - 100%, olive - 80-99%, yellow - 60-80% and white <60%, the alignment used to prepare this figure can be found in Supplementary Data Sheet 5.

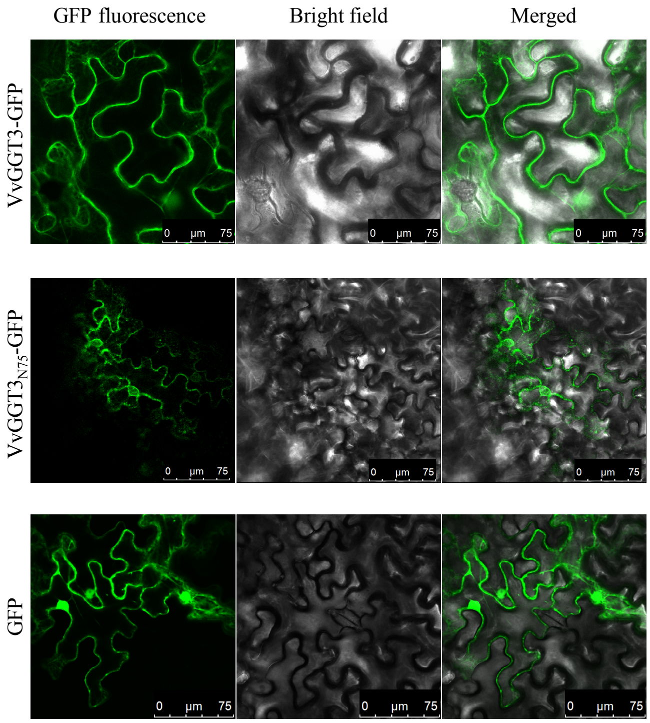

Supplementary Figure S4. Confocal images of *Nicotiana benthamiana* leaf cells transiently transformed with either the VvGGT3-GFP fusion protein **(Top row),** $VvGGT3_{N75}$-GFP fusion protein **(Middle row)** or GFP alone **(Bottom row)**. The merged images demonstrate that neither the full length (VvGGT3) nor the first 75 amino acids ($VvGGT3_{N75}$) localise to the vacuole as predicted by phylogenetic analysis. The free GFP localises to the nucleus and cell membranes as described in Ohkama-Ohtsu et al., 2007b.

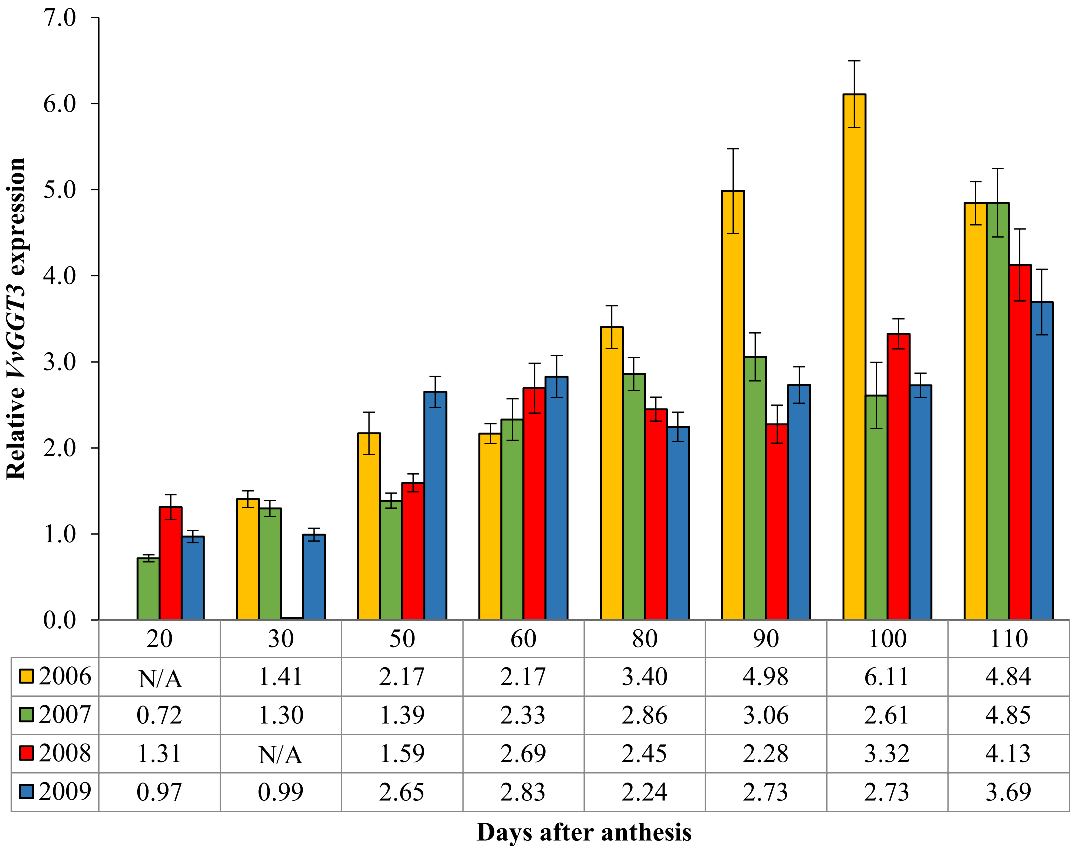

	20	30	50	60	80	90	100	110
2006	N/A	1.41	2.17	2.17	3.40	4.98	6.11	4.84
2007	0.72	1.30	1.39	2.33	2.86	3.06	2.61	4.85
2008	1.31	N/A	1.59	2.69	2.45	2.28	3.32	4.13
2009	0.97	0.99	2.65	2.83	2.24	2.73	2.73	3.69

Supplementary Figure S5. The mean relative abundance of *VvGGT3* in grape berries throughout development was studied in the 2006, 2007, 2008 and 2009 growing seasons. For each time-point, the phenologcal stages mostly represented E-L 30, 31, 32, 35, 36, 36, 37 and 38, respectively (Coombe, 1995). Veraison in each season took place approximately 50 to 60 days after anthesis (daa). The mean level at 20 daa across all growing seasons was set to 1. Data not available at 20 daa in the 2006 growing season and excluded at 30 daa in the 2008 growing season (N/A). The geometrical means of *VvActin* and *VvEF1α* as reference genes were used as a normalisation factors, n=3 technical replicates, means ± SEM. The mean relative abundance from each season was used as biological replicates to prepare Figure 4B.

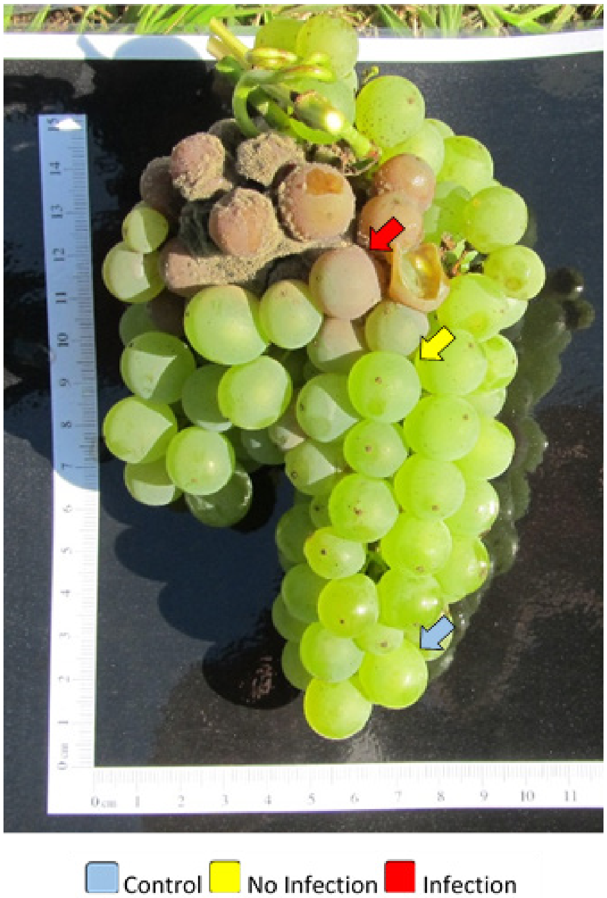

Supplementary Figure S6. Image of a *Botrytis cinerea* infected grape bunch. For *VvGGT3* transcript accumulation, we isolated berries from infected bunches that had (i) no sign of infection (Control), (ii) berries on infected bunches immediately adjacent to infected berries but did not show sign of infection (No Infection) and (iii) berries that showed signs of Botrytis infection, but were not fully infected (Infection).
